# Cation‐Assisted Lithium‐Ion Transport for High‐Performance PEO‐based Ternary Solid Polymer Electrolytes

**DOI:** 10.1002/anie.202016716

**Published:** 2021-05-04

**Authors:** Jaschar Atik, Diddo Diddens, Johannes Helmut Thienenkamp, Gunther Brunklaus, Martin Winter, Elie Paillard

**Affiliations:** ^1^ Helmholtz Institute Münster, IEK-12 Forschungszentrum Jülich GmbH Corrensstr. 46 48149 Münster Germany; ^2^ MEET Battery Research Center University of Münster Corrensstr. 46 48149 Münster Germany; ^3^ Politecnico di Milano Department of Energy Via Lambruschini 4 20156 Milan Italy

**Keywords:** batteries, ionic liquids, lithium, lithium transport, polymer electrolytes

## Abstract

*N*‐alkyl‐*N*‐alkyl pyrrolidinium‐based ionic liquids (ILs) are promising candidates as non‐flammable plasticizers for lowering the operation temperature of poly(ethylene oxide) (PEO)‐based solid polymer electrolytes (SPEs), but they present limitations in terms of lithium‐ion transport, such as a much lower lithium transference number. Thus, a pyrrolidinium cation was prepared with an oligo(ethylene oxide) substituent with seven repeating units. We show, by a combination of experimental characterizations and simulations, that the cation's solvating properties allow faster lithium‐ion transport than alkyl‐substituted analogues when incorporated in SPEs. This proceeds not only by accelerating the conduction modes of PEO, but also by enabling new conduction modes linked to the solvation of lithium by a single IL cation. This, combined with favorable interfacial properties versus lithium metal, leads to significantly improved performance on lithium‐metal polymer batteries.

## Graphical abstract

Ionic liquids (ILs) allow improvement of the ionic conductivity of ternary PEO‐based solid polymer electrolytes. However, the lack of Li‐ion coordination of these plasticizers and the addition of extra ions results in a low Li‐ion conductivity. An oligo(ethylene oxide)‐based IL was synthesized to overcome these limitations and enable additional transport modes, resulting in a high Li‐ion conductivity.

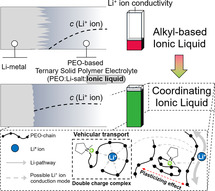

## Introduction

The current electrification of transport and decarbonizing of electricity production pushes battery researchers to explore new battery systems beyond the currently and dominant lithium‐ion technology.^[1]^ Lithium metal is considered as the “holy grail” of negative electrodes due to its ultrahigh theoretical specific capacity (i.e. 3860 mAh g^−1^ vs. 372 mAh g^−1^ for state‐of‐the‐art graphite electrodes) and its very low standard reduction potential (−3.04 V vs. standard hydrogen electrode).^[2]^ Many challenges, however, limit a widespread deployment of rechargeable lithium‐metal batteries (LMBs), including inhomogeneous electrodeposition of lithium metal, leading to the formation of high surface area lithium (HSAL).[^3^, ^4^] The formation of HSAL in the form of dendrites present serious safety hazards, especially when highly flammable organic liquid electrolytes are utilized as dendrites might readily penetrate the separator and induce internal short circuits.[^3^, ^4^, ^5^] Therefore, alternative electrolytes with high ionic conductivity, yet with better mechanical, chemical, electrochemical, and thermal stability than liquid electrolytes must be developed to facilitate the adoption of LMBs at large scale. Poly(ethylene oxide) (PEO)/lithium salt complexes are promising candidates in this respect, although they have been studied for more than 50 years as solid polymer electrolytes (SPEs).[^6^, ^7^] The good mechanical stability of cross‐linked PEO systems, their wide electrochemical stability window (ESW) and the excellent ability of PEO chains to dissolve lithium salts are all suitable for a use in LMBs.^[6]^ PEO/lithium bis(trifluoromethanesulfonyl)imide (LiTFSI) salt complexes are able to reach ionic conductivities up to 10^−3^ S cm^−1^ at 80 °C, but the ionic conductivity drops below useful values at low temperatures,^[8]^ (i.e. ≈10^−4^ S cm^−1^ at 40 °C for the best amorphous complexes^[9]^), thereby preventing the use of “dry” PEO‐based SPEs at room temperature so far. A feasible solution consists in adding plasticizers to increase ionic mobility.^[10]^ Ionic liquids (ILs) are promising in this respect due to their ultra‐low vapor pressure, broad ESW, high thermal and chemical stability and non‐flammability.^[11]^ PEO/Li salt/IL ternary solid polymer electrolytes (TSPEs) can reach ionic conductivities of up to 10^−3^ S cm^−1^ at 40 °C with ILs based on *N*‐alkyl‐*N*‐methylpyrrolidinium (Pyr_1,x_TFSI, *x* being the number of carbon atoms in the longer alkyl chain).^[12]^ Molecular dynamic (MD) simulations by Diddens et al.[^13^, ^14^, ^15^] showed that the enhanced ionic conductivity results from the increased segmental mobility of PEO chains. As for binary PEO/salt complexes, the actual lithium transport still occurs mainly along the polymer chains[^13^, ^14^, ^15^] rather than involving solvation sphere including the anion or oxygen from other polymer chains, since PEO preferentially solvates Li^+^ cations via consecutive oligo(ethylene oxide) units. Li^+^ ion‐transport modes can be differentiated between “structural” transport, such as that occurring along the PEO chain by exchanges of Li^+^ ion solvating units in its dynamic solvation sphere, and “vehicular” transport, corresponding to the transport of the Li^+^ ion with its solvation sphere before any exchange occurs (more predominant, for instance, in oligo(ethylene oxide) liquid electrolytes).^[16]^ Alkyl pyrrolidinium‐based ILs do not interact much with Li^+^ ions since the anion is far less coordinating than PEO. As a result, PEO/Li salts conductive paths are “diluted” by the IL addition and the Li‐PEO interaction is in some cases increased, slowing down Li^+^ ion transport.[^13^, ^14^, ^15^] This, together with the addition of IL ions that do not participate in the Li^+^ ion transport, results in a reduced lithium transference number (*t*_Li_^+^), which limits the potential performance gains when employing these electrolytes in LMBs.

**Figure 1:**
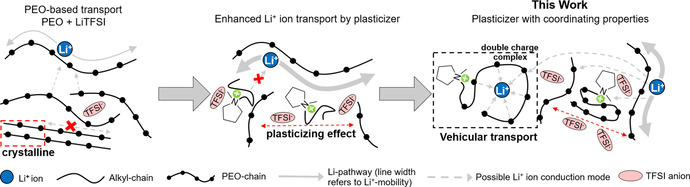
Illustration of the acceleration of PEO Li^+^ ion conduction modes by state‐of‐the‐art alkyl‐based IL plasticizers and, as proposed and investigated here, the enabling of new conduction modes via the coordinating Pyr_1,(2O)7_TFSI IL.

Therefore, to decouple Li^+^ ion transport, at least partially, from the segmental mobility of PEO chains, solvating ILs are a priori more suitable for enabling further transport modes as illustrated in Figure 1 (e.g. structural or vehicular modes involving IL species in Li^+^ ion solvation spheres). In particular, the presence of solvating cations could advantageously accelerate Li^+^ ion transport via the formation of complexes with two positive charges overall. We reported previously on improved interaction of Li^+^ ion with short oligo(ethylene oxide) chains‐substituted pyrrolidinium‐based ILs in liquid electrolytes. The data suggested that the first oxygen from the nitrogen center is not interacting with lithium and that longer oligo(ethylene oxide) chains are necessary for reaching the full solvation of Li^+^ ions by a single cation considering that Li^+^ ion complexes have a preferred coordination number of 6.[^17^, ^18^, ^19^] Aiming at the Li^+^ ion solvation by a single pyrrolidinium cation and thus enabling vehicular transport by the formation of charged complex with two positive charges, we report here on the synthesis of *N*‐methyl‐*N*‐oligo(ethylene oxide) pyrrolidinium TFSI with a median oligo(ethylene oxide) chain length of seven repeating units (noted Pyr_1,(2O)7_TFSI; Supporting Information). We compared the designed IL with Pyr_1,4_TFSI in terms of physiochemical properties and Li^+^ ion‐IL interaction in binary liquid electrolyte as well as in TSPEs. Lithium mobility and its conduction mode in TSPEs were analyzed by electrochemical measurements, pulsed field gradient nuclear magnetic resonance (PFG‐NMR) spectroscopy and MD simulation. Practical improvements were then verified by testing the rate performance and cycling stability of Li∥Li and LiFePO_4_ (LFP)∥Li cells.

## Results and Discussion

### Physicochemical properties and Li^+^ ion coordination in liquid binary Pyr_1,(2O)7_TFSI‐based and PEO‐based TSPEs

For the fundamental understanding of the coordination process and its evidence, Pyr_1,(2O)7_TFSI was characterized as liquid binary electrolyte LiTFSI:IL (mol:mol) prior to membrane preparation and characterization.

**Figure 2:**
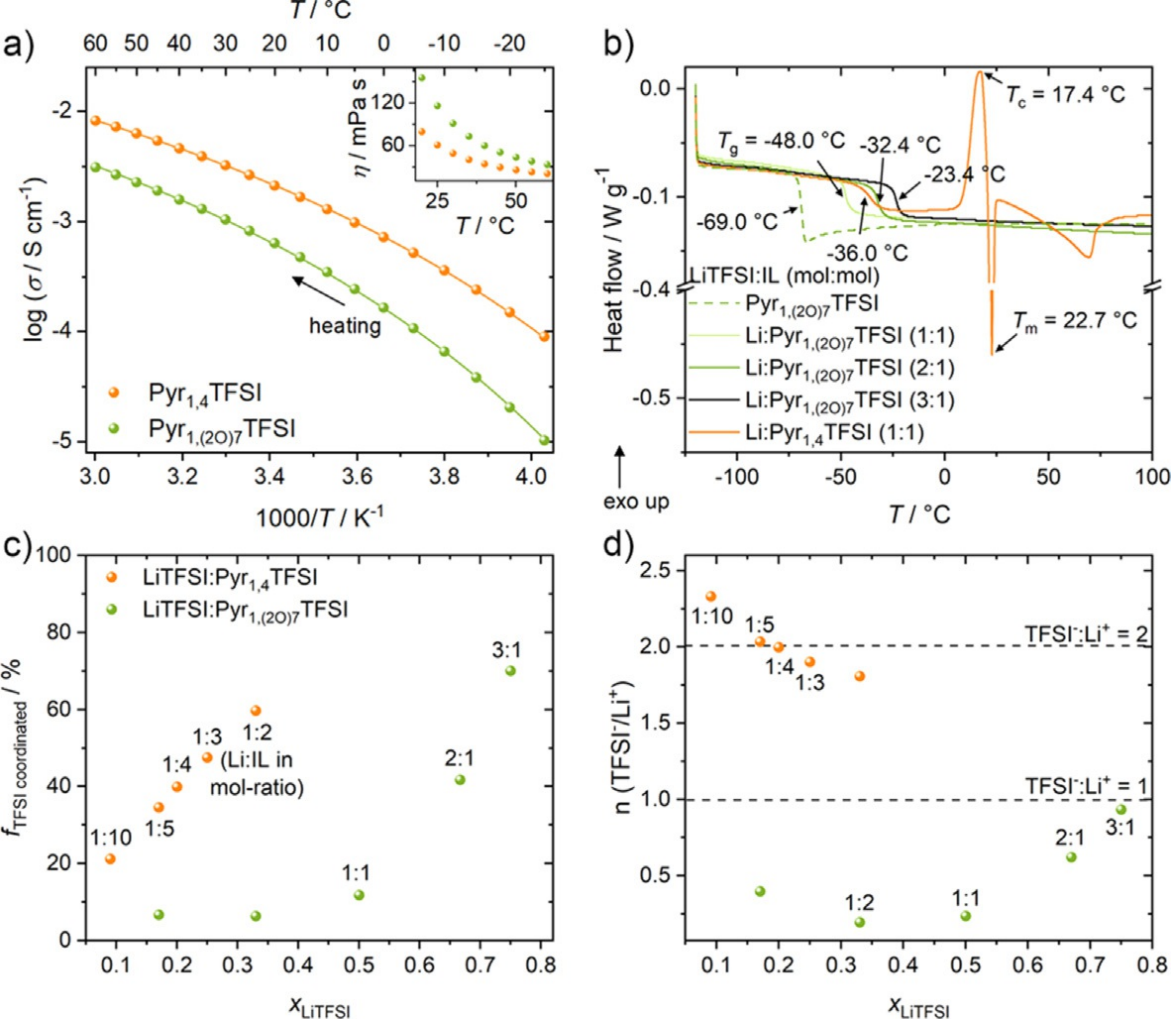
Characterization of Pyr_1,(2O)7_TFSI and Pyr_1,4_TFSI as pure component and binary electrolyte formulations. a) Ionic conductivity of the pure ILs from −25 to 60 °C with viscosity in the inset. b) DSC measurements for different IL‐based electrolyte compositions. c) Fraction of coordinated TFSI (*f*_TFSI_ coordinated) calculated from Raman spectra at different molar ratios of LiTFSI:x_1_ (x_1_=Pyr_1,(2O)7_TFSI or Pyr_1,4_TFSI) (mol:mol) at room temperature. d) Calculated average number of TFSI^−^ ions in the Li^+^ coordination sphere.

The ionic conductivity and the viscosity of the pure Pyr_1,(2O)7_TFSI is compared with that of Pyr_1,4_TFSI (Figure 2 a and inset). DSC thermograms of the liquid electrolytes with LiTFSI are shown in Figure 2 b. The large size of Pyr_1,(2O)7_TFSI leads expectedly to a higher viscosity and consequently to a lower ionic conductivity than Pyr_1,4_TFSI.

Interestingly though, when mixed with LiTFSI at high mole fractions, the binary mixtures are all amorphous (Figure 2 b), whereas the 1:1 LiTFSI:Pyr_1,4_TFSI complex exhibits two melting transitions corresponding to crystalline phases.^[20]^ As for the glass transition point (*T*_g_), although it increases with the salt content as a result of increased interactions and decreased ion mobility, the 1:1 mixture still exhibits a *T*_g_ of −48 °C that is remarkably low for such a high salt concentration and hinting at a well‐preserved ion mobility as compared with the Pyr_1,4_TFSI complex that exhibits a 12 °C higher *T*_g_, even though Pyr_1,4_TFSI has a *T*_g_ of −86 °C^[21]^ (vs. ‐69.0 °C for Pyr_1,(2O)7_TFSI). At high temperature, TGA results show a slightly earlier onset of Pyr_1,(2O)7_TFSI weight loss. The TSPE 20:2:1_O7_, cross‐linked or linear, exhibits a thermal stability very close to that of Pyr_1,4_TFSI TSPEs (Supporting Information, Figure S2a).

Raman spectroscopy allows deriving the Li^+^ ion solvation in TFSI‐based electrolytes, since the TFSI^−^ bands are sensitive tools for analyzing the ionic coordination. The band at 742–744 cm^−1^ is attributed to “free” (uncoordinated) TFSI^−^ whereas a band at 747–750 cm^−1^ is associated with coordinated TFSI.^[22]^ The relative ratio of the two bands (for the corresponding Raman spectra, Figure S1a) allows quantifying the amount of TFSI^−^ coordinated to Li^+^ (Figure 2 c) and deriving the number of coordinating TFSI^−^ per Li^+^ solvation shell (Figure 2 d). For LiTFSI mole fractions lower than 0.33 (i.e. corresponding to the first high melting crystalline phase of the LiTFSI/Pyr_1,4_TFSI system^[20]^), there is only a small fraction of coordinated TFSI^−^ anion in the LiTFSI/ Pyr_1,(2O)7_TFSI mixtures up to 1:1. In contrast, the Pyr_1,4_TFSI electrolytes show a linear increase of coordinated TFSI^−^ from 1:10 to 1:2 (a metastable liquid in the experimental conditions) where 60 % of TFSI^−^ anions are coordinated to Li^+^ ions. In terms of TFSI^−^ per Li^+^ ions coordination shell, the Pyr_1,4_TFSI electrolytes shows approximately two TFSI^−^ per Li^+^, similarly to previous reports,^[18]^ slightly decreasing at the highest concentrations but limited to ≈1.7 (TFSI^−^/Li^+^) for the 1:2 electrolyte. For Pyr_1,(2O)7_TFSI, on the other hand, even at a very high 1:1 mole ratio we calculate only 0.24 TFSI^−^ per Li^+^, which shows that most Li^+^ do not have any TFSI^−^ in their solvation spheres (only 12 % coordinated TFSI^−^) and are thus mostly coordinated by the cations. The number of TFSI^−^ per solvation sphere only significantly increases above 1:1 molar ratio (i.e. above the saturation of the oligo(ethylene oxide) solvating sites) with a rather linear trend, corroborating our initial hypothesis that seven repeating units are required for the pyrrolidinium side chain to fully solvating one Li^+^ ion.

**Figure 3:**
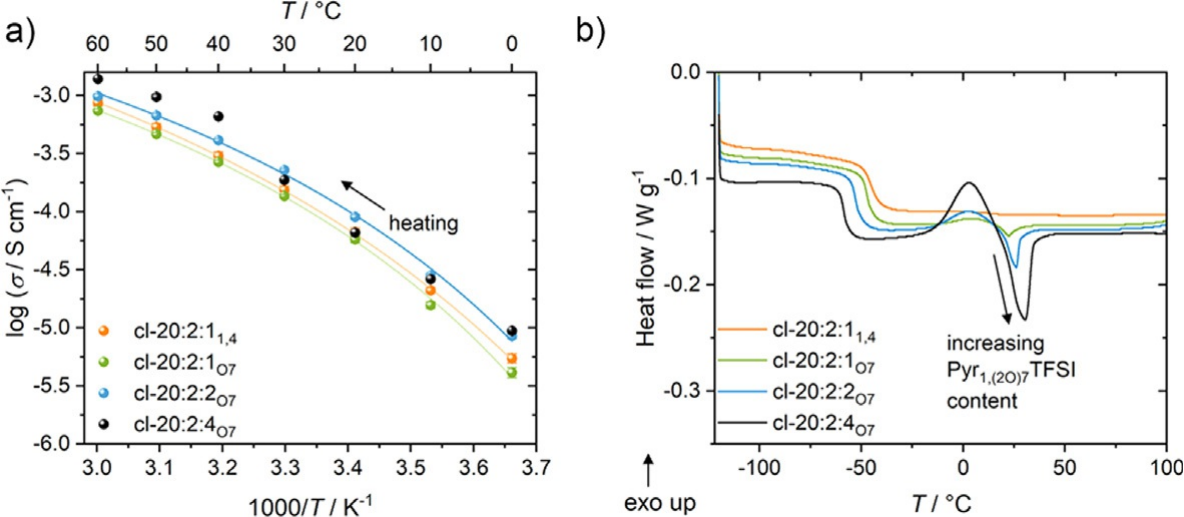
a) Ionic conductivities of different cross‐linked TSPE compositions (dots) with VFT fitting (line). b) DSC thermogram of the first heating curve of quenched cross‐linked PEO‐based TSPEs between −120 to 100 °C at 5 K min^−1^.

The effect of Pyr_1,(2O)7_TFSI cationic solvation on the physicochemical properties of TSPE membranes was then investigated. The preparation of the TSPEs is described in the Supporting Information. In the following, the TSPEs are labeled using their PEO:LiTFSI:IL molar stoichiometry (the number for PEO corresponds to the number of repeating ‐(CH_2_)_2_O‐ units) and the IL used is indicated as subscript (O7 for Pyr_1,(2O)7_TFSI and 1,4 for Py_1,4_TFSI). The prefix (cl‐) is used for TSPEs that have been cross‐linked by UV irradiation. Cross‐linking is typically introduced used to improve the mechanical properties of “dry” and plasticized polymer electrolytes, preventing creeping under pressure, while also hindering membrane crystallization.^[23]^ The ionic conductivities and DSC thermograms of cross‐linked polymer membranes (those of the linear TSPEs can be found in Figure S2b,c) are shown in Figure 3 a,b. A higher IL content increases crystallinity, even though TSPEs exhibit only very limited crystallinity (very small peaks compared to the *T*_g_ steps). In fact, only the membrane with the highest IL content (cl‐20:2:4) shows a transition in the ionic conductivity curves (while still exhibiting a well‐maintained ionic conductivity (for detailed values see the Supporting Information, Table S1) below the melting transition). Cross‐linking allows increasing the IL content in the TSPEs to lift the ionic conductivities even higher (6.6×10^−4^ S cm^−1^ and 1.4×10^−3^ S cm^−1^ at, respectively 40 °C and 60 °C for cl‐20:2:4_O7_). Even though higher ionic conductivities can be reached for the cl‐20:2:4_O7_ membrane and IL phase separation might be favorable to improve the performance and wetting^[24]^ in LFP∥Li cells, TSPEs with lower IL content were used in the following to avoid partial crystallization at an operating temperature of 40 °C.

### Lithium‐ion mobility and conduction modes in PEO‐based TSPEs

**Figure 4:**
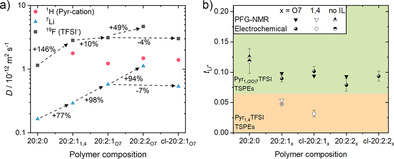
a) Self‐diffusion coefficients of different ionic species in polymer electrolytes (^1^H for pyrrolidinium‐based IL, ^7^Li for lithium ion, and ^19^F for TFSI^−^) measured by PFG‐NMR with an estimated error of ±2 % relative to calibration. Dashed arrows indicate the percentage increase between values. b) Overview of Li^+^ ion transference numbers determined from either electrochemical data or from PFG‐NMR for Pyr_1,4_TFSI‐, Pyr_1,(2O)7_TFSI‐based TSPEs, and for the 20:2:0 SPE. All samples were measured at 40 °C.

The mobility of each ionic species based on the self‐diffusion coefficients derived from PFG‐NMR experiments (Figure 4 a).

Switching from Pyr_1,4_TFSI to Pyr_1,(2O)7_TFSI in 20:2:1 TSPEs results in a moderate relative increase of TFSI^−^ mobility, with a significantly higher relative increase of lithium diffusion coefficient in (i.e. a 98 % increase from 0.292×10^−12^ to 0.578×10^−12^ m^2^ s^−1^), whereas the ion mobility of the larger pyrrolidinium cation is lower. This shows that the interaction of Li^+^ with the immobile PEO matrix is lowered and suggests that in TSPEs, IL cation‐Li^+^ complexes are formed assisting lithium‐ion transport. Cross‐linking does not affect considerably the results (decrease of less than 7 % for cl‐20:2:1_O7_ vs. the linear TSPE). Increasing the IL content to 20:2:2 yields a further increase of diffusion coefficients, in accordance with ionic conductivity. The Li^+^ transference numbers were estimated by using either the self‐diffusion coefficients or the “Bruce and Vincent” electrochemical technique^[25]^ (Figure S3a). The results (Table S2) vary slightly depending on the utilized method used but are consistent within the error margin as shown in Figure 4 b. By substituting Pyr_1,4_TFSI with Pyr_1,(2O)7_TFSI in 20:2:1 TSPE, a doubling of *t*_Li_^+^ can be reached at 40 °C (i.e. from 0.05±0.01 to 0.10±0.01) and even higher for the cross‐linked TSPEs. The increase of *t*_Li_^+^ is likely due to the enhanced solvation by the Pyr_1,(2O)7_^+^ cation, affording a similar *t*_Li_^+^ compared to the 20:2:0 “dry” SPE. Increasing the IL content in the 20:2:2 and cl‐20:2:2 membranes keeps *t*_Li_^+^ on a similar level to 20:2:1 but boosts the Li^+^ ion conductivity even higher because of the higher ionic conductivity.

To unravel the molecular lithium‐ion transport mechanism, MD simulations were performed. We extended our previous study^[19]^ by taking into account longer oligo(ethylene oxide) side chains and explicitly focusing on the correlated motion between Li^+^ and pyrrolidinium ions. In particular, we simulated TSPEs with oligo(ethylene oxide)‐based ILs with side chain lengths of one, four, and eight ethylene oxide monomers at a molar ratio of 20:2:1.

In the MD simulations, we note that 1.0 %, 6.2 % and 28.2 % of all lithium ions are not coordinated to PEO chains for 20:2:1_O1_, 20:2:1_O4_, and 20:2:1_O8_, respectively (*p*_IL_ in Table 1). This is in good agreement with the observations from Figure 2 c, reflecting that for sufficiently long oligo(ethylene oxide) side chains, the IL cations preferentially coordinate the Li^+^ ions. For 20:2:1_O1_ and 20:2:1_O4_, the values in Table 1 are slightly lower than reported previously for the corresponding systems with a concentration of 20:2:4 due to the lower amount of oligo(ethylene oxide)‐based IL.^[19]^

**Table 1:** Structural and dynamic quantities extracted from the MD simulations (Supporting Information).

System	*p*_IL_ [%]	*τ*_1_ [ns]	*τ*_R_ [ns]	*τ*_2_ [ns]	*τ*_3_ [ns]	*τ*_IL_ [ns]	*D*_Li_ (*N* → ∞) [×10^−11^ m^2^ s^−1^]
20:2:1_O1_	1.0	556	57	65	96	1	0.9
20:2:1_O4_	6.2	475	56	65	64	6	1.5
20:2:1_O8_	28.2	310	57	76	50	27	3.3

Key: percentage of lithium ions that are not coordinated to PEO (*p*_IL_), time scale a lithium ion requires to diffuse along the entire PEO chain it coordinates to (*τ*_1_), relaxation times of the average (*τ*_R_) and the bound (*τ*_2_) PEO segments, residence times of the lithium ions at a given PEO chain (*τ*_3_) or within the IL domain (*τ*_IL_); *D*_Li_ was computed by the transport model for the experimental condition *N* → ∞ (Supporting Information).

In conventional PEO‐based polymer electrolytes, three different transport mechanisms are generally identified:^[26]^ First, motion of a coordinated lithium ion along the backbone of a PEO chain, second, the cooperative motion of polymer segments with the coordinated Li^+^ ions, and third, the transfer of Li^+^ ions between two different PEO chains. Previously, we developed an analytical lithium‐ion transport model, in which the significance of each of the above mentioned transport mechanism is characterized by specific time scales *τ*_1_, *τ*_2_, and *τ*_3_, which can be extracted from MD simulations. Here, *τ*_1_ is the time a given Li^+^ requires to explore the PEO chain by diffusing along its backbone, *τ*_2_ denotes the relaxation time of the polymer segments bound to Li^+^ ions, while *τ*_3_ indicates the average residence time of a Li^+^ ion at a given chain. The particular benefit of our model comprises the extrapolation of the *D*_Li_ to the experimentally important limit *N* → ∞. Further details on the transport model, the extraction of the time scales and the calculation of the *D*_Li_ can be found in the Supporting Information.

From Table 1, we observe that *τ*_1_ decreases with increasing oligo(ethylene oxide) side chain length of the IL cation, that is, the Li^+^ ion motion along the backbone becomes more efficient as the Li^+^ ions become progressively coordinated to the IL cations (see discussion of *p*_IL_), rendering the ions that remain coordinated to PEO more mobile. Interestingly, the segmental dynamics expressed by the Rouse time *τ*_R_ ^[27]^ (all monomers) and *τ*_2_ (bound monomers) are approximately constant for all side chain lengths. In this context, we demonstrated that ILs act as plasticizers in PEO membranes, which increases the dynamics of the lithium ions attached to the polymer chains as the polymer motion itself is enhanced by the plasticizer.[^13^, ^14^, ^15^] The *τ*_2_ values from Table 1 suggest that such a plasticizing effect is comparable for all simulated ILs, and that increasing the oligo(ethylene oxide) chain length only marginally increases *τ*_2_. We observe that *τ*_3_ decreases by almost a factor of two when going from 20:2:1_O1_ to 20:2:1_O8_, illustrating that, for sufficiently long oligo(ethylene oxide) chains at the IL cation, Li^+^ is structurally and dynamically decoupled from the PEO chains, resulting in a shorter polymer residence time. This change is accompanied by a concomitant increase of the average time *τ*_IL_ during which Li^+^ ion is only coordinated by IL ions (either cations or anions) from about 1 to 27 ns when comparing 20:2:1_O1_ to 20:2:1_O8_ (Table 1). These observations demonstrate that the ether‐functionalized IL cations decouple the lithium ions from the PEO chains and serve as molecular shuttles in this way.

As in our previous work,^[19]^ we used our transport model to compute *D*_Li_ in the limit of long chains (Table 1; Supporting Information). We observe that when going from the essentially non‐coordinating IL cation with one ethylene oxide monomer only (i.e. 20:2:1_O1_) to 20:2:1_O4_ or 20:2:1_O8_, *D*_Li_ increases by factors of 1.7 and 3.7, respectively. Although the absolute *D*_Li_ values in Table 1 cannot be directly compared to the experimental PFG‐NMR values due to higher temperature in the MD simulations, these findings nonetheless clearly confirm the trends for *D*_Li_ observed in Figure 4 a, where similar factors are found.

So far, we focused on the self‐diffusion of the lithium ions. However, for quantities such as the ionic conductivity or the transference number (when determined via the electrochemical method), the cooperative motion of distinct ions—as expressed by dynamical ion correlations—is important as well.[^28^, ^29^, ^30^, ^31^] The ionic conductivity *σ* can then be derived from equilibrium simulations via the expression[^28^, ^29^, ^30^, ^31^]

$$\sigma =\underset{\Delta t\to \infty }{lim}\frac{e{}^{2}}{6V\Delta tk{}_{B}T}\overset{M}{\underset{i=1}{\sum }}\overset{M}{\underset{j=1}{\sum }}z{}_{i}z{}_{j}\left\langle \Delta r{}_{i}\left(\Delta t\right)\Delta r{}_{j}\left(\Delta t\right)\right\rangle$$

where *M* is the total number of ion pairs in the simulation box with volume *V*, e is the elementary charge, Δ*t* the observation or lag time, *k*_B_ *T* the thermal energy, *z_i_* and *z_j_* the (integer) charge numbers and Δ***r**_i_* and Δ***r**_j_* the displacement vectors of ions *i* and *j*. The terms with *i*=*j* correspond to the self‐diffusion contribution, whereas the dynamical ion correlations are expressed by the terms with *i*≠*j*. A typical example for the impact of these correlations includes the cooperative motion of cation‐anion pairs, which decreases *σ* because the product *z_i_* *z_j_* is negative and the dot product ⟨Δ***r**_i_*Δ***r**_j_*⟩ positive due to the cooperative motion of the ions. In the present case, however, one would expect that the IL cations carry the lithium ions over larger distances, resembling a shuttling mechanism and thus an increase of the ionic conductivity and the Li^+^ transference number. Unfortunately, extracting absolute values for ionic conductivities or transference numbers from MD data is challenging for statistical reasons, especially for a polymer host with large intrinsic relaxation times (this contrasts the single‐ion diffusion, which can be averaged over individual ions).

**Figure 5:**
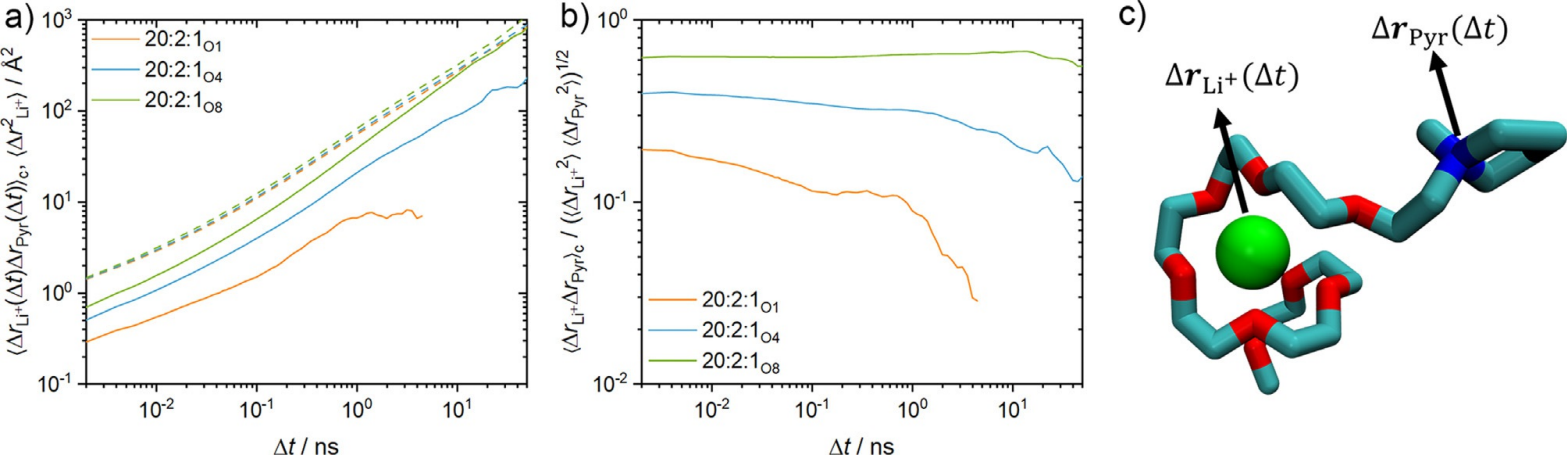
Correlation ⟨Δ***r***_Li_+Δ***r***_Pyr_⟩_c_ of the displacement vectors of Li^+^/IL cation pairs that are initially coordinated (i.e. with a center‐of‐mass separation less than 7 Å) as a function of time. The dashed lines indicate the averaged MSDs of the lithium ions. b) The same correlation normalized by the geometric mean of the MSDs of lithium ions and IL cations. c) Scheme of the correlated motion of a Li^+^‐Pyr_1,(2O)8_^+^ complex.

To nonetheless probe the correlated motion of IL cations and Li^+^ ions, we computed ⟨Δ***r***_Li_+(Δ*t*)Δ***r***_Pyr_(Δ*t*)⟩_c_ for all initially coordinated pairs of lithium ions and IL cations (i.e., when their initial center‐of‐mass separation was not larger than 7 Å) as a function of Δ*t* (Figure 5 a, compare to illustration in Figure 5 c). The mean squared displacement (MSD) ⟨Δ***r*** 2Li+ ⟩ averaged over all lithium ions is also shown for comparison (dashed curves). We find that ⟨Δ***r***_Li_+Δ***r***_Pyr_⟩_c_ increases substantially when increasing the oligo(ethylene oxide) side chain length and becomes even comparable to the lithium ion MSD for a length of eight monomers. This suggests that the correlated motions between Li^+^ ions and IL cations significantly contributes to the overall lithium‐ion conductivity, as observed from the experimentally determined transference numbers of 20:2:1_1,4_ and 20:2:1_O7_ (Figure 4 b).

Figure 5 b also shows ⟨Δ***r***_Li_+Δ***r***_Pyr_⟩_c_ normalized by the geometric mean of the MSDs of the lithium ions and the IL cations. Within this representation, a perfectly correlated motion between initially coordinated ions of either species would result in a value of one. From Figure 5 b, we find values of up to 0.6 on short time scales for 20:2:1_O8_. Values below unity presumably occur due to the rotation of the Li^+^/IL cation complexes and the internal degrees of freedom of the side chain, nonetheless, the correlation is significant. This is also reflected by the fact, that the relative correlation for shorter oligo(ethylene oxide) chains is substantially lower. On larger time scales, the correlation diminishes due to the exchange of Li^+^/IL cation pairs. This decay is slowest for 20:2:1_O8_, reflected by an average pair lifetime of about 100 ns, followed by 20:2:1_O4_ (≈10 ns) and 20:2:1_O1_ (0.07 ns; Supporting Information).

Our simulations confirm our approach to employ functionalized pyrrolidinium cations with sufficiently long oligo(ethylene oxide) side chains, which can indeed carry Li^+^ ions over larger distances (nanometers), significantly enhancing both the single‐ion transport (*D*_Li_) and the cooperative motion between Li^+^ and IL cations in this way.

### Practical properties and electrochemical performance of cross‐linked TSPEs for Li∥Li and LFP∥Li cells

**Figure 6:**
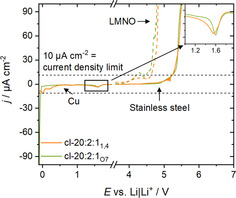
Anodic and cathodic stabilities of cross‐linked TSPE cl‐20:2:1_O7_ and cl‐20:2:1_1,4_ at 0.025 mV s^−1^ on Cu WE (cathodic sweep), stainless steel and LNMO (anodic sweep). Inset shows the first reduction peak on copper at 40 °C. Dashed lines show the current density limit.

Electrochemical requirements for any electrolyte, either polymer or liquid, are its ability to facilitate sufficient ion transport at the electrode|electrolyte interface while being subjected to as little electrochemical degradation (anodic/cathodic) as possible. The cross‐linked samples, cl‐20:2:1_O7_ and cl‐20:2:1_1,4_ were explored in terms of ESW on copper for cathodic stability and on stainless steel and LiNi_1.5_Mn_0.5_O_4_ (LNMO) for anodic stability (Figure 6). An important property of an electrolyte includes its partial electrochemical reduction at the Li|electrolyte interface to ensure an effective solid electrolyte interphase (SEI)^[32]^ formation, to inhibit further electrolyte reduction and to enable stable long‐term cycling. The first reductive peak appears at ≈1.6 V (vs. Li|Li^+^), which is commonly observed.^[33]^ The peak appears for both TSPEs, indicating a negligible influence of oligo(ethylene oxide) side chain (Figure 6 insert) and no significant peaks are seen before lithium electrodeposition. On the anodic scan, the voltamperograms are comparable with overlapping curves and an anodic stability up to 5.2 V (vs. Li|Li^+^) close to the ESW of pure Pyr_1,(2O)7_TFSI (5.0 V vs. Li|Li^+^; Figure S3a) as well, considering a 0.1 mA cm^−2^ limit in both cases. Nevertheless, common lithium‐metal polymer batteries (LMPB) composite electrodes, such as LFP, exhibit higher surface area and a vastly different surface reactivity and the TSPEs stability was thus probed using a high voltage spinel LNMO composite electrode as well. It resulted in a similar oxidative stability for both TSPEs of ≈4.6 V (vs. Li|Li^+^).

**Figure 7:**
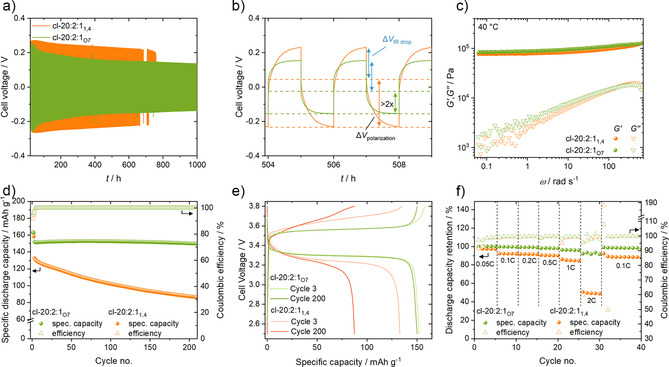
a) Voltage profiles of Li∥Li cells cycled with cl‐20:2:1_O7_ and cl‐20:2:1_1,4_ at 0.1 mA cm^−2^ at 40 °C with b) a focus on three consecutive cycles. The dashed lines indicate the IR drop and end of step voltages. c) Evolution of storage (*G*′) and loss (*G*′′) moduli at 40 °C with angular frequency at 0.2 % strain. d) Specific discharge capacity of LFP∥Li cells at 40 °C cycled at 1 C (0.05 C for the first 2 cycles). e) Voltage profiles for the 3^rd^ and 200^th^ cycles. f) Normalized discharge capacity with increasing discharge C‐rate. Charge step was kept at 0.1 C with different discharge C‐rates as shown. 1 C is equivalent to ≈0.2 mA cm^−2^.

The TSPEs performance in terms of galvanostatic lithium plating/stripping, which depends strongly on their interfacial properties toward Li metal, was investigated in symmetrical Li∥Li cells and is reported in Figure 7 a for the crosslinked TSPEs and in Figure S6 for 20:2:0 and 20:2:1_O7_). The decrease in voltage over time is likely due to some evolution of SEI resistance during cycling. At a current density of 0.1 mA cm^−2^, the cell voltage is reduced by ≈35 % for cl‐20:2:1_O7_ compared to cl‐20:2:1_1,4_ (0.15 vs. 0.23 V) at 40 °C and the effect is even more marked at 60 °C (Figure S4a,b). A closer look at the voltage profile in Figure 7 b shows that the voltage can be divided into two main contributions. The IR drop (Δ*V*_IR drop_) at the beginning of each step is assigned to the initial resistance, dominated by the SEI, the contact and electrolyte resistance. The second part is an asymptotical voltage increase during each step, which is attributed to the establishment of ionic concentration gradients (Δ*V*_polarization_) that become steeper and steeper until a steady‐state is reached (or the lithium concentration reaches 0 at the plated electrode, at which point fast dendrite growth becomes inevitable^[34]^). Since gradient formation depends on Li^+^ ion mobility, they are strongly influenced by *t*_Li_^+^_._ The differences in terms of initial IR drop are rather limited and do not show any obvious trend which was also verified by a following cell impedance evolution either during galvanostatic cycling or at rest (Figure S4c,d). A significantly lower voltage plateau due to a lower Δ*V*_polarization_ can be observed for cl‐20:2:1_O7_ (Figure 7 b dashed lines). It is half compared to cl‐20:2:1_1,4_ (that does not allow reaching steady‐state in one hour). These improvements result in no short circuit after 1000 h for cl‐20:2:1_O7_. In fact, cl‐20:2:1_O7_ also allows maintaining higher steady‐state currents and, as a result, obtaining more homogenous plating onto Cu (Figure S7). To verify that the prevention of a short circuit does not result from increased mechanical stability, dynamic shear rheometry experiments were performed (Figure 7 c). The determination of the linear viscoelastic range (LVE) is reported in Figure S5a,b. The two TSPEs cl‐20:2:1_1,4_ and cl‐20:2:1_O7_ show comparable storage and loss moduli values. It indicates that the mechanical stability differences have, at best, a minor influence on the Li cycling behavior. In comparison to linear TSPEs, a further advantage of the cross‐linking is the much improved elasticity and limited deformation at low frequencies as seen Figure S5c,d.

Finally, the effect of the improved Li^+^ mobility of the new TSPE on LMBs performance was verified by cycling in LFP∥Li cells. Figure 7 d compares the cycling stability of LFP∥Li cells with either a cross‐linked Pyr_1(2O)7_TFSI TSPE or a Pyr_1,4_TFSI analog. The initial Coulombic efficiency (CE) increased from 91.6 % for cl‐20:2:1_1,4_ to 95.7 % for cl‐20:2:1_O7_ and from 99.93 % to 99.96 % in the following cycles, which would have a highly beneficial effect on cycle life with a smaller Li metal electrode. Beside the excellent CEs, the specific discharge capacities are higher for cl‐20:2:1_O7_ (151 mAh g^−1^ in the 5^th^ cycle,). Most importantly, the long‐term capacity retention is considerably improved. After 200 cycles, the cl‐20:2:1_O7_ cell retains 99.3 % of its initial capacity (referred to the 5^th^ cycle) vs. 67.2 % for cl‐20:2:1_1,4_). The reasons for this can be attributed to the slower Li^+^ ion transport in cl‐20.2:1_1,4_. Although LFP is known for a very flat voltage plateau at 3.4 V (vs. Li|Li^+^),^[35]^ the plateau of the cl‐20:2:1_1,4_ cells is more sloped than that of the cl‐20:2:1_O7_ cell (Figure 7 e), which results in a lower capacity at 0.5C in the 3^rd^ cycle. In addition, the plateau becomes increasingly sloped as an effect of the increasing cell polarization over cycling which explains the capacity decay. It likely results from the formation of HSAL at a current density of ≈0.1 mA cm^−2^, as seen in Li∥Li experiments and, over cycling, to a degraded transport at the Li|electrolyte interface. In contrast, the cl‐20:2:1_O7_ cell exhibits flat plateaus with constant cell voltage over cycling. Due to the increasingly slower lithium‐ion transport in the cl‐20:2:1_1,4_ cell, the cut‐off voltage is reached faster and faster, and the capacity decays. This is also visible in the discharge rate performance of the cells (Figure 7 f). The capacity retention already decreases to 92.4 % from 0.05 C to 0.1 C (related to 1^st^ cycle) for cl‐20:2:1_1,4_ compared to 99.8 % with cl‐20:2:1_O7_. Even at 1 C, a capacity retention of 96.8 % is reached for cl‐20:2:1_O7_. At 2 C, the differences in capacity retention are even stronger with 50.0 % for cl‐20:2:1_1,4_ vs. 93.5 % for cl‐20:2:1_O7_. This clearly illustrates the improved rate performance induced by Pyr_1,(2O)7_TFSI. In both cells, the initial specific discharge capacity at 0.1 C can be reached after the rate test, showing that at constant charge rate, the cells (i.e. the lithium‐metal electrode) were not overly affected by the discharge (i.e. lithium electrodissolution). It shows, however, that the much faster lithium transport results in much higher rate performance capability of the LMPB cells, regardless of the degradation of the lithium‐metal interface.

## Conclusion

Pyr_1,(2O)7_TFSI was specifically designed with an oligo(ethylene oxide) side chain on its pyrrolidinium cation to overcome the limitations of *N*‐alkyl‐*N*‐alkyl pyrrolidinium‐based ILs in terms of *t*_Li_^+^ when used as plasticizers for PEO‐based TSPEs. This IL is not only highly promising for formulating super‐concentrated binary liquid electrolytes, since it allows reaching a 3:1 (LiTFSI:Pyr_1,(2O)7_TFSI mol:mol) liquid composition, but it also enables PEO‐based polymer membranes with far higher performance than Pyr_1,4_TFSI analogues, although having similar physicochemical properties. In particular, the cross‐linked TSPE cl‐20:2:1_O7_ possesses a similar ionic conductivity as the state‐of‐the‐art cl‐20:2:1_1,4_ electrolyte but exhibits a Li^+^ ion conductivity three times higher with a *t*_Li_^+^ of 0.10±0.01 at 40 °C (vs. 0.03±0.01 for cl‐20:2:1_1,4_). This increase in *t*_Li_^+^ reflects the excellent solvating properties of Pyr_1,(2O)7_TFSI that enable fast lithium‐ion transport, especially via enabling an additional “vehicular” transport mode since the IL cation is able to solvate one Li^+^ ion. Our MD simulations confirmed the cooperative motion of Li^+^ ions and IL cations both via a simplified model for the Li^+^ ion transport and an explicit analysis of dynamical ion correlations. These insights show that, the use of ionic shuttle molecules opens up new avenues to improve the ion‐transport properties of TSPEs. Practically, thermally stable and elastic membranes allow significantly higher rate performance of LFP∥Li LMPB cells. Long‐term cycling of symmetrical Li∥Li and LFP∥Li cells show that the faster lithium transport results in well‐maintained cell performance with no deterioration over cycling of the lithium transport, contrary to what happens with Pyr_1,4_TFSI‐based TSPEs in the same conditions. In the latter case, the increasingly slower lithium transport over cycling is attributed to the evolution of the lithium‐metal interface as slow lithium transport favors the formation of HSAL. On the contrary, a 99.3 % capacity retention is reached with cl‐20:2:1_O7_ (vs. 67.2 % for cl‐20:2:1_1,4_) after 200 cycles. This demonstrates that faster lithium transport results not only in higher power capability but also in safer and longer living LMPB cells.

## Conflict of interest

The authors declare no conflict of interest.

## Supporting information

As a service to our authors and readers, this journal provides supporting information supplied by the authors. Such materials are peer reviewed and may be re‐organized for online delivery, but are not copy‐edited or typeset. Technical support issues arising from supporting information (other than missing files) should be addressed to the authors.

Supplementary
