# Supplementary material for: Cation‐Assisted Lithium‐Ion Transport for High‐Performance PEO‐based Ternary Solid Polymer Electrolytes
Source: Angew Chem Int Ed Engl. 2021 May 4;60(21):11919–27. doi: 10.1002/anie.202016716 (PMC8252488; doi:10.1002/anie.202016716)
Supplement: Supplementary file 1 — Supplementary [file ANIE-60-11919-s001.pdf]

Angewandte Chemie
Eine Zeitschrift der Gesellschaft Deutscher Chemiker

Supporting Information

Cation-Assisted Lithium-Ion Transport for High-Performance PEO-based Ternary Solid Polymer Electrolytes

Jaschar Atik, Diddo Diddens, Johannes Helmut Thienenkamp, Gunther Brunklaus, Martin Winter, and Elie Paillard**

anie_202016716_sm_miscellaneous_information.pdf

Table of Contents

1 Experimental procedures....1

1.1 Materials....1

1.2 Synthesis of $Pyr_{1,(2O)7}$TFSI....1

1.3 Preparation of polymer electrolyte and composite cathode....2

1.4 Cell assembly....2

1.5 Spectroscopic investigation....3

1.6 Thermal & physicochemical analysis....3

1.7 Electrochemical investigations....3

2 Supplementary Figures and Tables....5

3 Molecular Dynamics Simulation Details & Extraction of Transport Model Parameters....13

4 References....16

5 Author Contributions....16

1 Experimental procedures

1.1 Materials

Poly(ethylene glycol) mono methyl ether (mPEG350MME) (M_w ≈ 350 g mol^{-1}, Sigma Aldrich), pyridine (anhydrous, 99.8%, Sigma Aldrich), 4-toluenesulfonyl chloride (TsCl) (≥ 98%, Sigma Aldrich), sodium hydrogen carbonate (99%, Alfa Aesar), *N*-butyl-*N*-methylpyrrolidinium bis(trifluoromethylsulfonyl)imide ($Pyr_{1,4}$TFSI) (≥ 99.0%, Merck), hydrochloric acid (37%, Sigma Aldrich), sodium iodide (anhydrous, ≥ 99%, Sigma Aldrich), sodium thiosulfate pentahydrate (99.5%, ACROS Organics™), *N*-methylpyrrolidine (≥ 98.0%, Sigma Aldrich), lithium bis(trifluoromethanesulfonyl)imide (LiTFSI) (battery grade, 3M), *N*-methyl-2-pyrrolidone (NMP) (99.5%, Sigma Aldrich), poly ethylene oxide (PEO) (M_w = 5.000.000 g mol^{-1}, Sigma Aldrich), benzophenone (99%, Sigma Aldrich), activated charcoal (decolorizing, Sigma Aldrich), polyvinylidene difluoride (PVdF) (Solef®), acetone (HPLC grade, Fisher Chemical), isopropyl alcohol (HPLC grade, Fisher Chemical), diethyl ether (HPLC grade, Fisher Chemical), dichloromethane (HPLC grade, Fisher Chemical), $LiFePO_4$ (LFP) (Süd-Chemie AG), Super C65 (Imerys), lithium metal (50 µm, Albemarle), molecular sieve (3 Å, Sigma Aldrich).

1.2 Synthesis of $Pyr_{1,(2O)7}$TFSI

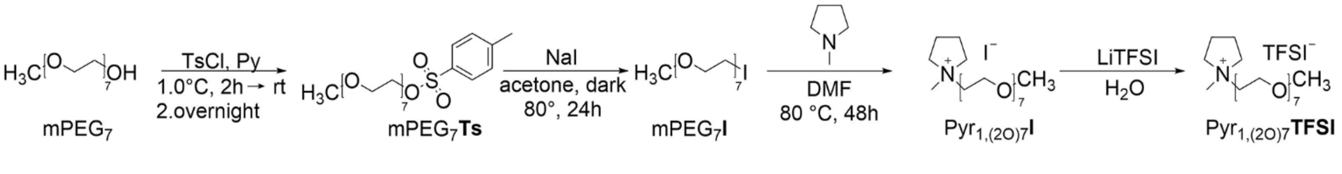

Scheme S1. Synthesis path for $Pyr_{1,(2O)7}$TFSI

$mPEG_7Ts$: The general procedure was taken from Ganapatibhotla *et al.* [1] and adapted to the pyrrolidinium-based ionic liquid (IL) with different workup and synthesis procedure for a higher scale (here 50 g) and optimizing the overall yield to 58%. mPEG350MME (32.2 mL, 100 mmol, 1.00 eq.) was set for 2h under vacuum to remove any oxygen impurities and mixed with anhydrous pyridine (16.1 mL, 200 mmol, 2.00 eq.). The solution was cooled down to 0 °C. TsCl (24.4 g, 128 mmol, 2.25 eq.) in anhydrous DCM (300 mL) was added in portion to the slightly amber-colored solution. The mixture was warmed back to room temperature (RT) and stirred overnight. The solution was washed with saturated $NaHCO_3$ solution (3x 80 mL), 5% aqueous HCl solution (3x 80 mL) and deionized water (3x 80 mL). The organic layer was dried over Na_2SO_4, filtered and concentrated under reduced pressure. After solvent evaporation, the liquid was dried furthermore at 70 °C for 24 h under reduced pressure to remove the excess of TsCl and solvent impurities. $mPEG_7Ts$ (55.2 g, quantitative) was obtained as slightly amber-colored, viscous compound. This mixture was used without

further purification. ^{1}H NMR (400 MHz, [D$_2$]CH$_2$Cl$_2$) δ / ppm = 7.85 (d, J = 7.9 Hz, **2H**, aryl ring), 7.35 (d, J = 7.9 Hz, **2H**, aryl ring), 4.10 (t, J = 4.6 Hz, **2H**, CH$_2$OTs), 3.70 - 3.43 (m, **28H**, CH$_2$C*H*$_2$O), 3.30 (s, **3H**, OCH$_3$), 2.41 (s, **3H**, Ar-CH$_3$). Spectra can be found in Figure S8.

mPEG$_7$I: mPEG$_7$ tosylate (51.9 g, 105 mmol, 1.00 eq.) was dissolved in anhyd. acetone (400 mL). NaI (47.2 g, 315 mmol, 3.00 eq.) was added in the dark under vigorous stirring. The mixture was stirred for under reflux for 24 h. The solvent was evaporated under reduced pressure and DCM (150 mL) and deionized water (130 mL) were added. The mixture was vigorously stirred for 30 minutes. The organic layer was washed with 5% aq. $Na_2S_2O_3$ solution (3x 70 mL), satd. $NaHCO_3$ solution (3x 70 mL) and additional deionized water (3x 70 mL). Solvent was evaporated and the product was dried under reduced pressure at 80 °C. mPEG$_7$I (39.4 g, 74%) was obtained as a yellow viscous liquid. ^{1}H NMR (400 MHz, [D$_2$]CH$_2$Cl$_2$) δ / ppm = 3.71 (t, J = 6.5 Hz, **2H**, C*H*$_2$CH$_2$I), 3.79 - 3.34 (m, **27H**, CH$_2$C*H*$_2$O), 3.31 (s, **3H**, OCH$_3$), 3.26 (t, J = 6.5 Hz **2H**, CH$_2$C*H*$_2$I).

Pyr$_{1,(2O)7}$I: *N*-methylpyrrolidine was purified by distillation prior to synthesis. *N*-methylpyrrolidine (15.3 mL, 147 mmol, 1.70 eq.) was added to mPEG$_7$I (39.4 g, 87.5 mmol, 1.00 eq.) in anhydrous DMF (40 mL) and stirred for at 80 °C for 48 hours. After cooling to RT, the solution was added slowly to cold Et$_2$O (0 °C). The upper Et$_2$O-layer was decanted off and the product was washed six times with fresh Et$_2$O (6x 50 mL). The last solvent impurities were evaporated under reduced pressure and dried at 70 °C overnight. Pyr$_{1,(2O)7}$I (37.6 g, 80%) was obtained as a dark red viscous liquid. ^{1}H NMR (400 MHz, [D$_2$]CH$_2$Cl$_2$) δ / ppm = 3.96 - 3.90 (m, **2H**, N$^+$C*H*$_2$CH$_2$-), 3.84 - 3.68 (m, **6H**, CH$_2$C*H*$_2$O and N$^+$C*H*$_2$CH$_2$-), 3.66 - 3.61 (m, **2H**, N$^+$CH$_2$C*H*$_2$O), 3.59 - 3.48 (m, **20H**, CH$_2$C*H*$_2$O), 3.46 - 3.41 (m, **2H**, N$^+$C*H*$_2$CH$_2$O), 3.27 (s, **3H**, OCH$_3$), 3.22 (s, **3H**, N$^+$CH$_3$), 2.26 - 2.11 (m, **4H**, N$^+$CH$_2$C*H*$_2$-)

Pyr$_{1,(2O)7}$TFSI: Pyr$_{1,(2O)7}$I (37.6 g, 70.2 mmol, 1.00 eq.) was dissolved in deionized water (150 mL) and LiTFSI (40.3 g, 140 mmol, 2.00 eq.) was added in portion and stirred overnight. The product was extracted with DCM (3x 50 mL) and washed with deionized water (6x 75 mL). Crude product was purified by activated charcoal (0.1 g activated charcoal per 1.0 g IL) in deionized water (100 mL) and was stirred at 70 °C overnight. This step was repeated until the compound was completely colorless. The product was extracted with DCM and then dried under stirring at 10^{-7} mbar for five days. Impurities of water can be removed by storing the IL over molecular sieve with reaching water level below 20 ppm determined by Karl-Fischer titration. Colorless oil was obtained (47.5 g, 98%). Polydispersity index: 1.07, Ion chromatography: c(I$^-$), c(Cl$^-$) < 0.1 ppm, ^{1}H NMR (400 MHz, [D$_2$]CH$_2$Cl$_2$) δ / ppm = 3.96 - 3.89 (m, **2H**, N$^+$C*H*$_2$CH$_2$-), 3.68 - 3.63 (m, **2H**, N$^+$CH$_2$C*H*$_2$O), 3.63 - 3.50 (m, **27H**, N$^+$C*H*$_2$CH$_2$- and CH$_2$C*H*$_2$O), 3.50 - 3.45 (m, **2H**, N$^+$C*H*$_2$CH$_2$O), 3.31 (s, **3H**, OCH$_3$), 3.10 (s, **3H**, N$^+$CH$_3$), 2.29 - 2.16 (m, **4H**, N$^+$CH$_2$C*H*$_2$-).

1.3 Preparation of polymer electrolyte and composite cathode

All components were dried prior to mixing as follow and stored afterwards in a dry room (dew point: < -65 °C): PEO was dried for at 10^{-7} mbar at 50 °C 4 days, LiTFSI at 150 °C for 2 days, all ILs at 80 °C under stirring for 5 days with following storage over molecular sieve and benzophenone at 50 °C for 2 days. Pyr$_{1,4}$TFSI was prior to the dry step washed several times with deionized water additional purified with activated charcoal according to the previous reported purification procedure. The polymer was prepared by mixing PEO with the conducting Li-salt LiTFSI, benzophenone and the IL in a mortar. Plasticized polymer was sealed in a pouch bag under vacuum and stored at 100 °C for two days. Afterwards, the polymer was hot-pressed between two Mylar foils (100 µm, silicon coated, PPI Adhesive Products GmbH) at 100 °C at 10 to 50 bar. Afterwards, ternary solid polymer electrolyte (TSPE) was cross-linked by induced polymerization with benzophenone as photoinitiator for five minutes each side by UV irradiation (UVACUBE 100, Dr. Hönle AG). The thickness of the TSPE was ≈100 µm for long term Li||Li and LFP||Li experiments and 200 µm for ionic conductivity and transference number determination. The composite cathode consisted of LFP (80.0 wt%), Super C65 (7.5 wt%), 5 wt% PVdF solution in NMP (7.5 wt% regarding PVdF) and TSPE in ratio of present electrolyte composition (5.0 wt%). All components were added to NMP and stirred for 24 h by magnetic stirring and afterwards heated up and stirred slowly at 60 °C for 2 h. The resulting mixture was coated on Al-foil (20 µm) and dried at elevated temperatures for 1 day. The composite cathode sheet was calendared and the punched electrodes were dried at 80 °C under reduced pressure ($1\cdot10^{-3}$ mbar) for 2 days. A resulting mass loading of 1.2 mg cm^{-2} was achieved for Pyr$_{1,4}$TFSI and Pyr$_{1,(2O)7}$TFSI containing ternary polymer composite cathodes.

1.4 Cell assembly

All cells were prepared in a dry room. The ionic conductivity measurements for TSPEs were performed in pouch bag cells sandwiching the polymer (thickness ≈ 200 µm) between two copper electrodes (area 4.84 cm^2). Lithium stripping/plating measurements and long-term cycling were performed in two electrode [2] coin cells (2032) with lithium and composite LFP electrodes (Ø = 12 mm, active mass loading ≈ 1.2 mg cm^{-1}) or two lithium electrodes in a symmetrical cell. The LSV for the determination of the electrochemical stability window (ESW) of TSPEs were performed in PAT-cells from EL-CELL® as two-electrode cell or as a three-electrode [2] cell set-up with stainless steel (Ø = 16 mm) or LiNi$_{0.5}$Mn$_{1.5}$O$_4$ (LNMO, Ø = 16 mm) as working electrode (WE) for anodic stability, copper (Ø = 16 mm) for cathodic stability and lithium as counter- (CE) and reference electrodes (RE). ESW measurements of liquid samples were measured in Swagelok® cells with copper (Ø = 12 mm) or polished platinum (Ø = 1 mm) as WE, lithium as CE and RE and FS2226 (3 layers, Freudenberg Performance Materials Holding SE & Co. KG) as separator with a 13 mm (140 µL) round shaped layer between CE and WE and 10 mm (70 µL) for reference contact.

1.5 Spectroscopic investigation

^{1}H samples were recorded on a BRUKER 400 AVANCE III HD instrument and PFG-NMR spectra on a BRUKER 200 AVANCE III HD instrument using a Diff50 probe. The PFG-NMR sample was sealed in glass tubes under vacuum and kept at 40 °C for 30 minutes prior to measurement. PFG-NMR data were acquired with a (double tuned ^{1}H/^{19}F & ^{7}Li) 5 mm coil at 40 °C. A 0.25M LiCl in H_2O solution, a 1% H_2O in D_2O with 0.1% $CuSO_4$ solution ("Doped Water") and a 3M KF in H_2O solution were utilized for external calibration (the error was less than 2%). The gradient strength was varied from 1400 to 2947 G cm^{-1} averaging up to 16 scans with a gradient pulse length *δ* of 1 ms and diffusion time Δ varied from 40 to 600 ms. The self-diffusion coefficients *D* of the lithium / fluorine / proton species were derived from a stimulated echo sequence ("diffSte") after fitting the attenuated signal amplitude to the Stejskal-Tanner equations, which describes the case of rather ideal ("free") isotropic diffusion:

$$I = I_0 \cdot \exp\left(-D\gamma^2\delta^2 g^2\left(\Delta - \left(\frac{\delta}{3}\right)\right)\right) \quad (1)$$

With *I* being the signal intensity, I_0 the initial signal in the absence of a magnetic field gradient and *γ* the gyromagnetic ratio. Data analysis for PFG-NMR was done with BRUKER Topspin 3.5 and BRUKER Dynamics Center 2.5. Data analysis for ^{1}H spectra were performed on MestReNova V.12.0. Lithium transference numbers from PFG-NMR data were calculated with the following equation[3]:

$$t_{\mathrm{Li^+,PFG-NMR}} = \frac{n_{\mathrm{Li^+}} \cdot D_{\mathrm{Li^+}}}{(n_{\mathrm{TFSI^-}} \cdot D_{\mathrm{TFSI^-}} + n_{\mathrm{Li^+}} \cdot D_{\mathrm{Li^+}} + n_{\mathrm{Pyr^+}} \cdot D_{\mathrm{Pyr^+}})} \quad (2)$$

Raman measurements were performed with a RAM II FT-Raman-module coupled to a VERTEX 70 FT-IR spectrometer with CW Nd:YAG laser with an excitation at 9395 cm^{-1}, power of 500 mW and a LN-Ge-diode detector. 1024 scans were measured for each spectrum with 1 cm^{-1} optical resolution. The detected range was 0 – 4000 cm^{-1}. The samples were sealed in NMR glass tubes under dry condition. The resulting spectra were fitted in a range of 720 to 770 cm^{-1} with OriginPro 2019 (OriginLab Corporation) using a Voigt function.

1.6 Thermal & physicochemical analysis

Thermogravimetric analysis (TGA) was performed on TA Instruments TGA Q5000. The samples were hermetically sealed inside an aluminum pan in a dry room and measured with a nitrogen flow of 10 mL min^{-1}. The sample was equilibrated at 30 °C, opened prior to measurement and heated up to 600 °C at 5 °C min^{-1}.
Differential scanning calorimetry (DSC) measurements were measured on a Q2000 by TA Instruments. The sample were hermetically sealed inside an aluminum pan in a dry room . Helium flow was set to 25 mL min^{-1} with a heating rate of 5 °C min^{-1} from -120 to 100 °C. Prior to the measurement, the sample was heated up to 100 °C for 20 minutes and quenched to -120 °C to obtain amorphous samples.

Rheological properties were determined on an Anton Paar Physica MCR 102 rheometer. For the determination of the viscosity of the liquid samples, a cone plate (Ø = 20 mm, angle = 0.5°, CP20-0.5, Anton-Paar) and a shear rate of 8000 s^{-1} were used for 5 s per measurement in 5 °C steps in a temperature range from 25 to 60 °C. Between each measurement, the temperature was kept for 30 minutes prior to measurement to anneal the liquid. The SPE were determined by using a parallel plate (Ø = 15 mm, PP15, Anton Paar). The amplitude sweep was done between 0.01 to 100% strain at 40 °C to detect the linear viscoelastic region (LVE). The membrane was heated up to 60 °C for 20 min and cooled down to 40 °C and annealed for 30 minutes prior to each measurement. For the frequency sweep, a range of 0.01 to 100 Hz was applied with 0.2% strain with the same temperature procedure. To ensure a constant pressure on the TSPE, a normal force of 5 N was applied during the whole measurement.

1.7 Electrochemical investigations

Ionic conductivity experiments were performed on an Autolab M204 (Metrohm AG) for polymer electrolytes and for liquid samples on a BioLogic MCS 10 with a WTSH 10 temperature control unit. The frequency range was from 1 Hz up to 1 MHz. The chosen electrode for liquid sample was a parallel plate platinized electrode on a borosilicate glass holder. The cell constant was calibrated prior to measurement with 0.01M KCl aqueous solution at 25 °C. Polymer samples were measured with copper (area: 4.84 cm^2) as electrode. The SPE thickness were measured prior to the measurement and afterwards and the average thickness was taken. The temperature range for polymer samples was 0 to 60 °C and for liquid samples -25 to 60 °C. The data were fitted with the Vogel-Fulcher-Tammann equation (eq.3). [4]

$$\sigma = \sigma_0 \cdot e^{-\frac{E_a}{R(T-T_0)}} \quad (3)$$

Potentiostatic and dynamic electrochemical measurements were performed on BioLogic VMP3 potentiostat-galvanostat-electrochemical impedance spectrometer. Linear sweep voltammetry (LSV) measurements were done as follows: PAT-cells from EL-CELL® were used for TSPE and Swagelok® for pure ILs using Li foil as counter and reference electrodes and various working electrodes at 0.025 mV s^{-1} from open current potential (E_{OCP}) to 6.5 V (*vs*. Li|Li^+) for the anodic stability and between E_{OCP} and -0.025 V (*vs*. Li|Li^+) for the cathodic stability at 40 °C.
Lithium ion transference number (t_{Li^+}) was determined by using a combined potentiostatic polarization and complex impedance measurement technique according to equation (4). [5]

$$t_{\mathrm{Li^+}} = \frac{I_{\mathrm{SS}}\left(\Delta V - I_0 R_{\mathrm{f,0}}\right)}{I_0(\Delta V - I_{\mathrm{SS}} R_{\mathrm{f,SS}})} \quad (4)$$

Symmetrical PAT-cell with two lithium electrodes (Ø = 16 mm) and TSPE (Ø = 18 mm) membranes were assembled. The cells were kept at open circuit for five days at the measured temperature prior to the measurement for allowing a good contact at the interfaces. Afterwards, impedance was measured in a frequency range between 100 mHz and 500 kHz and an amplitude of 10 mV voltage was then applied until the current reached a steady-state value (I_{SS}). The initial current (I_0) was calculated by the impedance data from the impedance spectrum acquired prior to polarization according to equation (5) with ΔV as amplitude voltage, $R_{el,0}$ as initial electrolyte resistance and $R_{f,0}$ as initial SEI resistance.

$$I_0 = \frac{\Delta V}{R_{el,0} + R_{f,0}} \quad (5)$$

Galvanostatic experiments were performed on a Maccor Series 4000 (excepted for the cells on which impedance measurement were taken that were cycled on a Biologic VMP3). Coin cells (2032) were used with a TSPE (thickness: 100 µm, Ø = 16 mm), lithium metal (Ø = 12 mm) as negative electrode (or in symmetrical Li||Li cells) and composite LFP (Ø = 12 mm, 1.2 mAh cm^{-1}). The first two cycles were cycled at 0.05C, followed by the indicated C-rate, named in figure caption. For the C-rate test, the charge C-rate was kept constant at 0.1C and varied for discharge.

2 Supplementary Figures and Tables

The calculation of the average number of TFSI⁻ coordinated to each Li⁺ (n) was done by following equation (6) where x is the mol fraction of the salt, f is the fraction of the coordinated TFSI⁻ and A corresponds to the peak areas. [6]

$$n = \frac{f_{TFSI^- \text{ corrdinated}}}{x_{salt}} = \frac{A_{TFSI^- \text{ coordinated}}}{x_{salt} \cdot A_{total}} \quad (6)$$

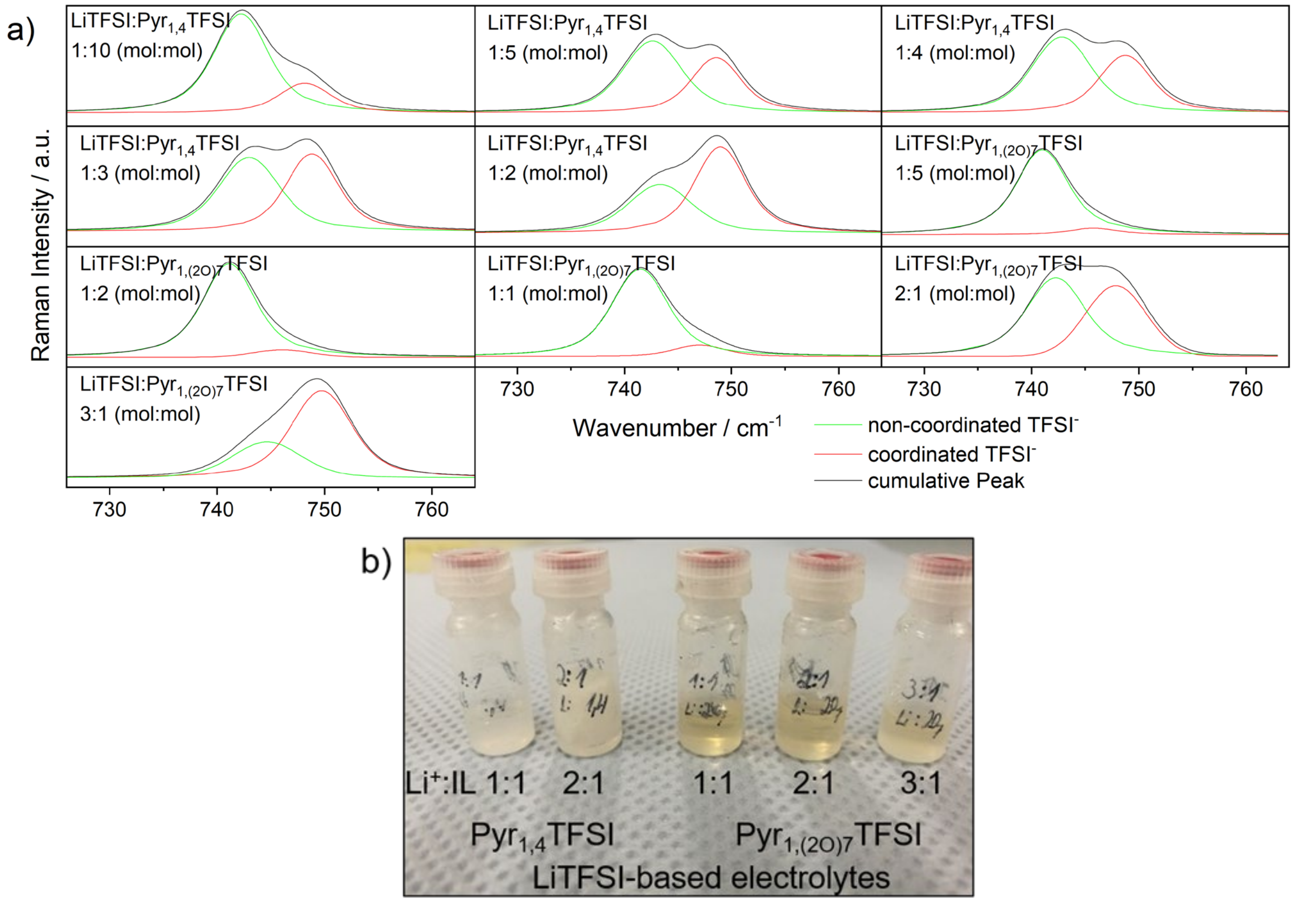

Figure S1. Characterization of the pure IL and LiTFSI-based electrolytes: a) Raman spectra of non-coordinated, coordinated and cumulative fitted TFSI⁻ band for different molar ratio of LiTFSI:IL for $Pyr_{1,4}$TFSI and $Pyr_{1,(2O)7}$TFSI at room temperature (RT), b) LiTFSI:IL-based electrolytes in mol-ratios cooled down from 90 °C to RT.

Figure S1b shows the appearance of the liquid electrolytes with different molar ratio of LiTFSI:IL. The samples were heated to 90°C and stirred for a maximum of 5 days and cooled down to room temperature. The $Pyr_{1,(2O)7}$TFSI electrolytes are completely liquid after cooling to room temperature for ratios up to 3:1. As for the $Pyr_{1,4}$TFSI-based electrolytes, the 1:1 starts to crystallize upon cooling and the 2:1 after few hours after staying in a metastable liquid state in agreement with literature [7] In the case of $Pyr_{1,(2O)7}$TFSI, the electrolytes remain in the liquid state after 4 weeks.

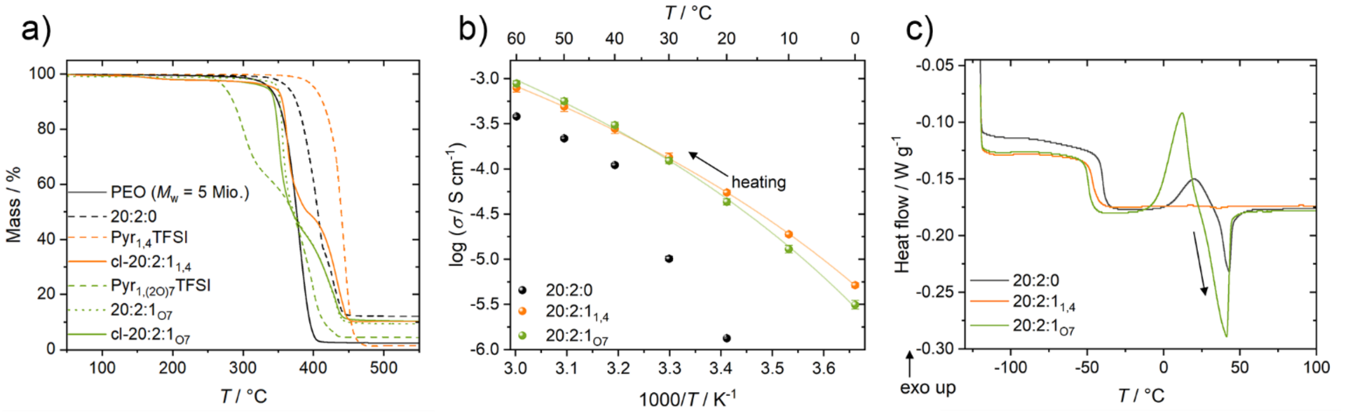

Figure S2. a) Thermogravimetric analysis of mass loss as a function of temperature of pure PEO, $Pyr_{1,(2O)7}$TFSI, specific linear/cross-linked (T)SPEs and $Pyr_{1,4}$TFSI as benchmark. Ionic conductivities of different b) linear TSPE compositions (dots) with VFT fitting (line). c) DSC thermogram of the first heating curve showing quenched linear PEO-based TSPEs between -120 to 100 °C with a heating rate of 5 K min^{-1}.

Figure S2b reports the ionic conductivities of linear TSPEs 20:2:1 *vs.* the 20:2:0 "dry" SPE (values can be found in Table S1). The latter exhibits a very low ionic conductivity below its melting point, seen as a transition from a linear Arrhenius to Vogel-Tammann-Fulcher (VTF) behavior. TSPE 20:2:1_{O7} allows reaching higher ionic conductivity than 20:2:$1_{1,4}$ at 40°C and above, while the contrary is visible at 30°C and below. Nevertheless, the DSC thermograms (Figure S2c) show that, in contrast to the binary LiTFSI:IL mixtures (main part), the linear TSPEs incorporating $Pyr_{1,(2O)7}$TFSI exhibit a higher fraction of crystalline phase (although still very low compared to the step corresponding to the T_g) than that with $Pyr_{1,4}$TFSI. The cold crystallization peak during the heating ramp is smaller than the melting peak for $Pyr_{1,(2O)7}$TFSI, indicating a partial crystallization occurring during cooling. However, the T_g of the $Pyr_{1,(2O)7}$TFSI linear TSPE is lower than that of the Pyr_{14}TFSI equivalent even though T_gs of semi-crystalline polymers are usually higher than in a purely amorphous polymer of similar composition.
The thermal stability of the IL, as shown in Figure S2a, $Pyr_{1,(2O)7}$TFSI is less stable than both $Pyr_{1,4}$TFSI and PEO (370 °C and 330 °C) with a weight loss starting at ≈ 250°C in N_2. The TSPE 20:2:1_{O7}, cross-linked or linear, exhibits a thermal stability very close to that of Pyr_{14}TFSI-based TSPEs, with no significant weight loss below 350°C.

Table S1. Ionic conductivities at 40 °C and glass transition/melting points from the DSC thermogram for selected TSPE compositions.

Membrane	Ionic conductivity (40 °C) / 10^{-4} S cm^{-1}	Glass transition (T_g / °C) / Melting point (T_m / °C)
20:2:0	1.1 ± 0.1	-39.8 / 43.1
20:2:$1_{1,4}$	2.8 ± 0.3	-46.3 / -
cl-20:2:$1_{1,4}$	3.0 ± 0.2	-45.8 / -
20:2:1 $_{O7}$	3.1 ± 0.2	-49.2 / 41.2
cl-20:2:1 $_{O7}$	2.7 ± 0.1	-47.5 / 22.6
cl-20:2:2 $_{O7}$	4.1 ± 0.1	-53.0 / 26.3
cl-20:2:4 $_{O7}$	6.6 ± 0.1	-58.6 / 30.4

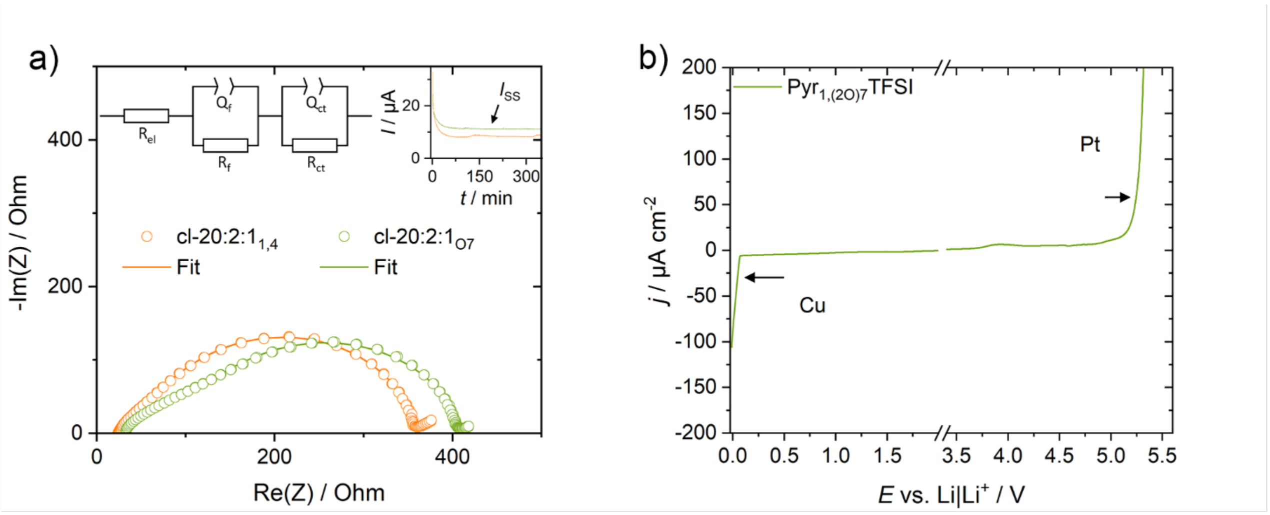

Figure S3. a) Example of Nyquist plots of Li||Li cells before and after (almost perfectly overlapping) applying a potentiostatic step (10 mV) until steady-sate for transference number determination (inset: corresponding chronoamperograms) to determine Li⁺-ion transference number (t_{Li^+}). b) Anodic and cathodic stabilities of $Pyr_{1,(2O)7}TFSI$ at 40 °C, WE: Pt (Ø = 1 mm) for anodic stability and copper (Ø = 12 mm) for cathodic stability, CE/RE: Li-metal, E_{OCP} to 6.5 V (*vs.* Li|Li⁺) for Pt and E_{OCP} to -0.025 V (*vs.* Li|Li⁺) for Cu.

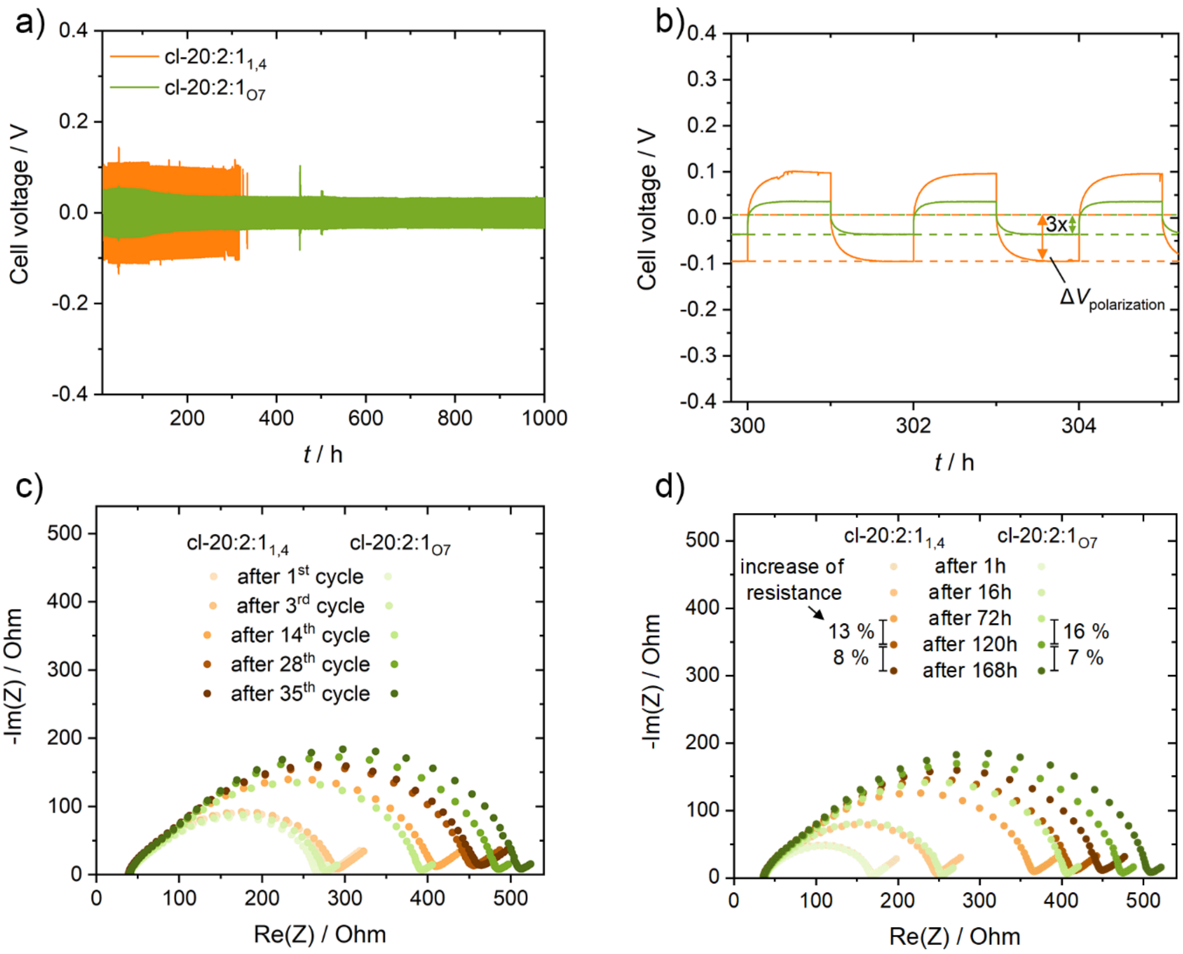

Figure S4. a) Voltage profiles of lithium platting/stripping experiment for long-term cycling stability on a Li||Li cell set-up with the benchmark electrolyte cl-20:2:1$_{1,4}$ and cl-20:2:1$_{O7}$ at 60 °C, b) for a chosen section of the voltage plots in the range of 300 to 305 h. Impedance evolution of Li||Li cell at 40°C c) During galvanostatic cycling of 0.1 mAh cm^{-2} at 0.1 mA cm^{-2} d) aging at open circuit after cell assembly.

Table S2. Overview of the t_{Li}^+ calculated from the diffusion coefficient determined by PFG-NMR and the those measured electrochemically. σ_{Li} was calculated from ionic conductivity measurements and electrochemical t_{Li}^+ values

Membrane	t_{Li}^+ by PFG-NMR (40°C)	t_{Li}^+ (40°C) electrochemical	$\sigma_{Li,calc}$ (40°C) / 10^{-5} S cm^{-1}
20:2:0	-	0.12 ± 0.02	1.3 ± 0.2
20:2:$1_{1,4}$	0.05	0.05 ± 0.01	1.4 ± 0.1
20:2:1_{O7}	0.10	0.09 ± 0.01	2.8 ± 0.2
cl-20:2:1 $_{O7}$	0.09	0.10 ± 0.01	2.7 ± 0.1
cl-20:2:1 $_{O7}$	-	0.12 ± 0.01@60 °C	8.9 ± 0.3@60 °C
cl-20:2:$1_{1,4}$	-	0.03 ± 0.01	1.0 ± 0.2
20:2:2 $_{O7}$	0.09	0.08 ± 0.01	3.6 ± 0.1

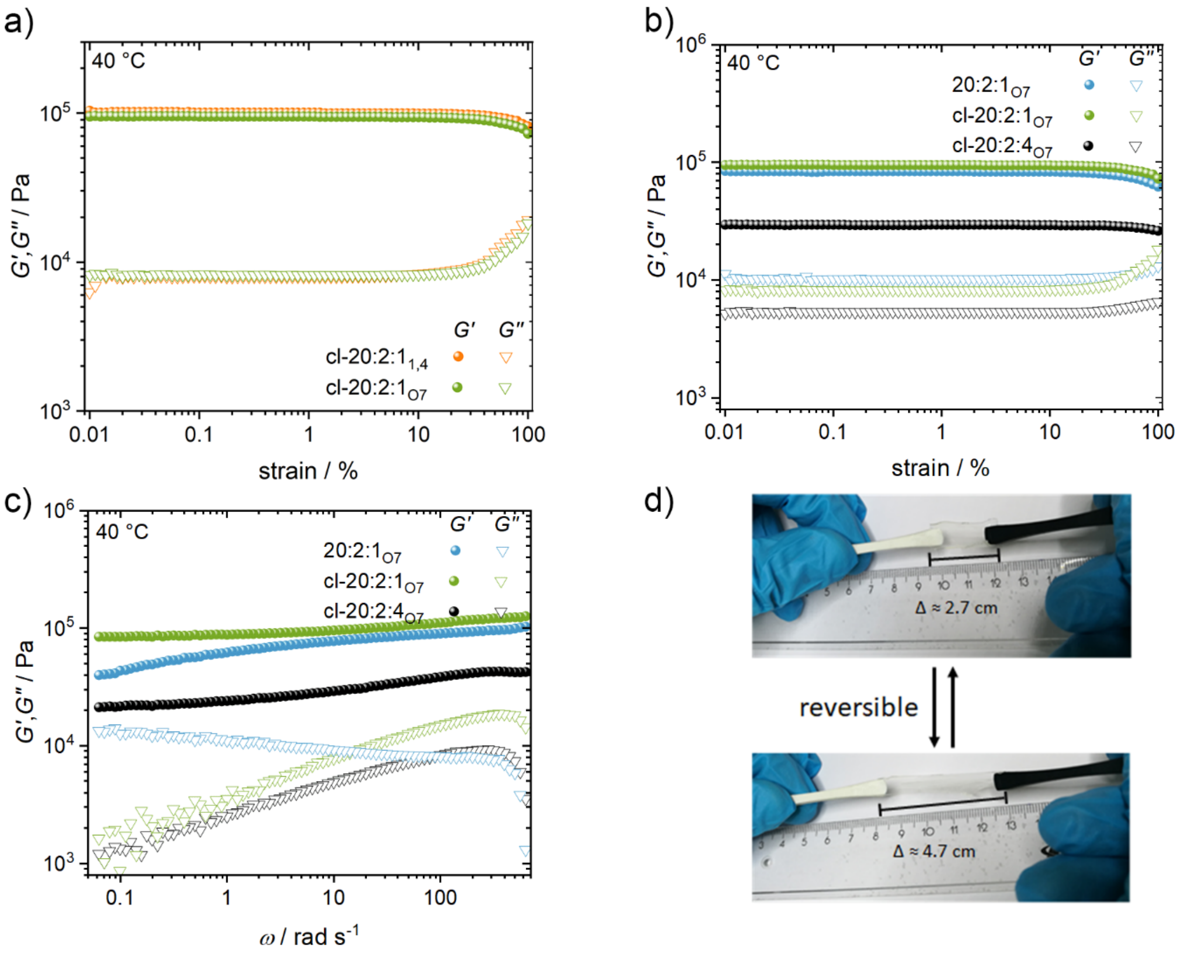

Figure S5. Storage (G') and loss moduli (G'') of different TSPEs. a) and b) Amplitude sweeps for the detection of the LVE region for linear and cross-linked samples with a constant frequency of 2 Hz and a strain range of 0.01 to 100% at 40 °C, c) Frequency sweeps at 0.1% strain in the range of 0.01 to 100 Hz at 40 °C, d) 200 µm thick cl-20:2:1$_{O7}$ under stretching condition at RT.

Dynamic shear rheometry was performed for determining the linear viscoelastic region (LVE) (figure S5a & S5b). The comparison between cross-linked and linear (figure S5c) TSPEs shows a lower overall storage modulus G' over the frequency range and a further decreasing values at low frequency values (as linear membrane creep under strain). Furthermore, the G'' loss modulus is increasing, showing a more less reversible behavior (i.e. deformation) also intensifying for low frequency ranges.

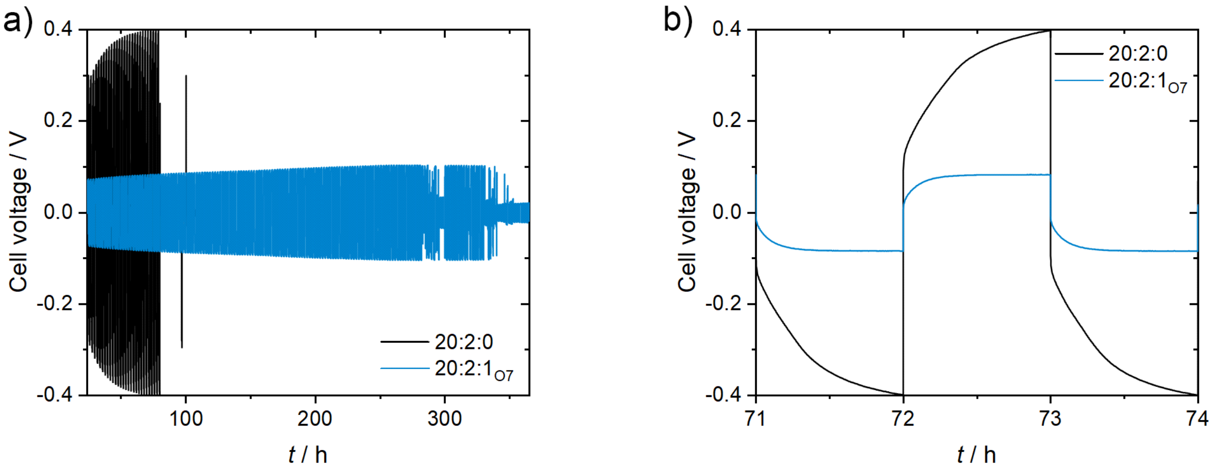

Figure S6. a) Voltage profiles of Li||Li cells cycled with 20:2:1_{O7} and 20:2:0 at 0.1 mA cm^{-2} at 40 °C with b) zoom on one cycle.

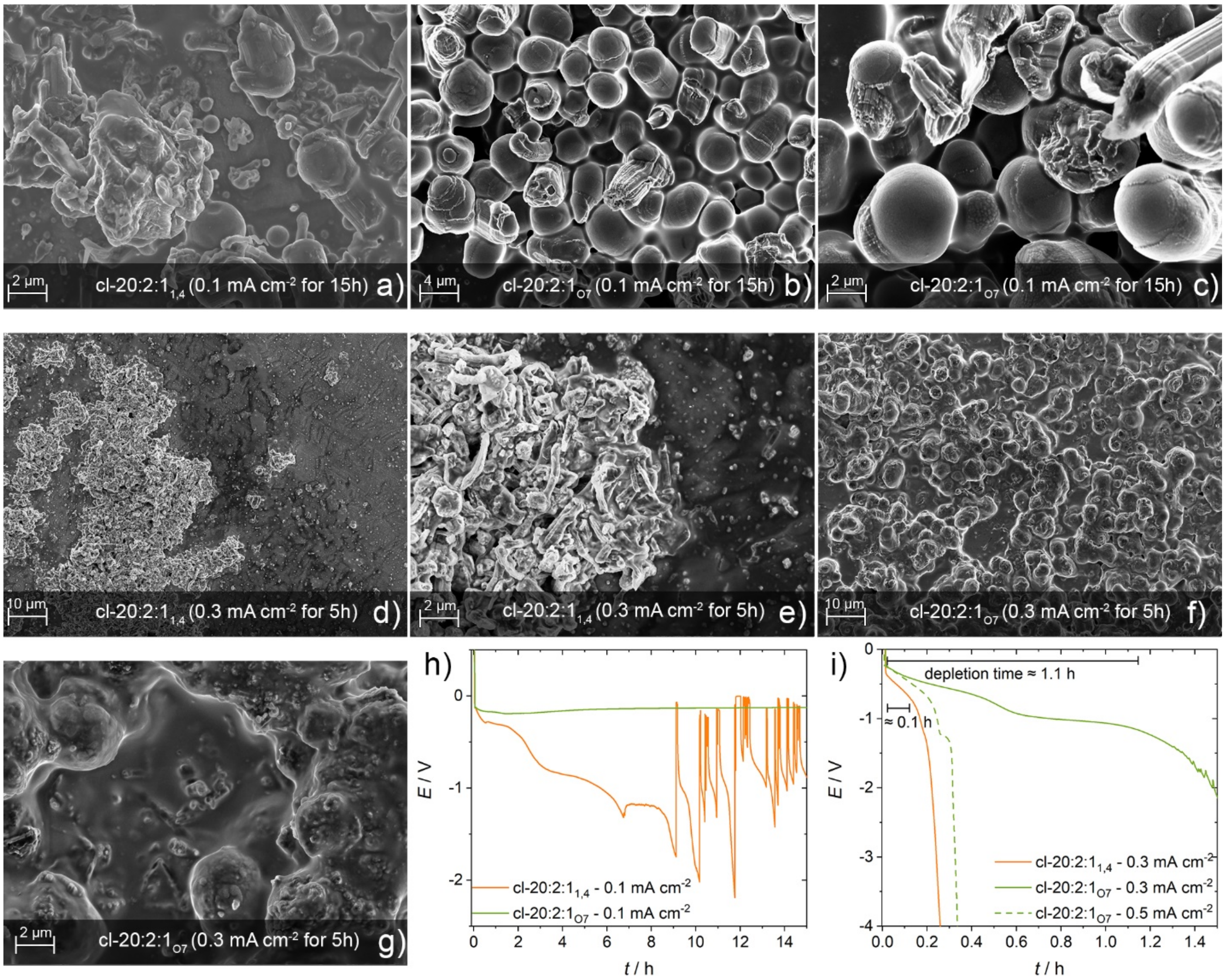

Figure S7. a-g) SEM pictures of lithium deposits onto Cu foil the current density and TSPE used are indicated in the images and the overall capacity was 1.5 mAh cm^{-2}. The electroplating were done at 40 °C. The corresponding voltage-time plot are shown in h) and i) for the different current densities.

The lithium electrodeposition onto Cu foil has been investigated with the TSPEs cl-20:2:1_{O7} and cl-20:2:$1_{1,4}$. Lithium was plated onto Cu at current densities of 0.1, 0.3 and 0.5 mA cm^{-2} for an overall capacity of 1.5 mAh cm^{-2} (or until the cut-off voltage of the equipment (i.e. -10V) was reached). After the experiments, the cells were opened and the TSPE membranes were swollen with DMC to allow their

removal from the lithium-plated copper and acquiring SEM images of the lithium deposits. The voltage profiles of the cells are shown in Figure S7 h) and i). Figure S7h shows that cl-20:2:1_{O7} allows reaching steady-state with a flat voltage curve for 15 h at a current density of 0.1 mA cm^{-2} whereas the cl-20:2:$1_{1,4}$ cell exhibits strong polarization after 2 hours, followed by the appearance of short-circuits. This cell polarization is linked to the fact that Li^+ transport in the electrolyte is not fast enough to sustain the applied current density. It is also observed for higher current densities (Figure S7i) where the polarization occurs even faster for cl-20:2:$1_{1,4}$ at 0.3 mA cm^{-2}. At 0.3 mA cm^{-2}, cl-20:2:1_{O7} allows sustaining the current density without marked polarization for more than 1 h. Starting with 0.5 mA cm^{-2}, high cell polarization in less than 1 h can also be observed for cl-20:2:1_{O7}. The morphologies of the lithium deposits are shown in Figure S7 a-g. The Li morphology for cl-20:2:1_{O7} at 0.1 mA cm^{-2} (Figure S7 b and c) shows that the lithium deposit is not flat but is rather dense and homogeneous over the whole surface with spherical shape formed Li metal, and do not show evidence of dendrites. In contrast, the deposit from the cl-20:2:$1_{1,4}$ cell is less homogeneous with some flat areas (copper) whereas others show inhomogeneous lithium deposits (some lithium was partly removed with the electrolyte membrane). It is likely an effect of lithium depletion since the lithium depletion favors the deposition onto lithium protrusions which might then evolve into dendrites. This is even more obvious at higher current densities with rod-shaped Li-metal dendrites seen in the cl-20:2:$1_{1,4}$ deposits. On the contrary, the plating at higher current densities with the cl-20:2:1_{O7} TSPE result in the same type of rather densely packed growth of lithium as for 0.1 mA cm^{-2}. However, at 0.3 mA cm^{-2} some growth of lithium into the TSPE occurred (with some polymer remaining with the deposits, as seen in Figure S7g) which can be explained as the plating was done until the voltage reached the cut-off after ≈ 3 h. Overall, the increase of the onset current that induces strong cell polarization is increased, showing a clear improvement in terms of lithium transport. A fully homogeneous plating onto Cu foil, however, would likely require addressing the lithium nucleation onto Cu, which is not the scope of this work.

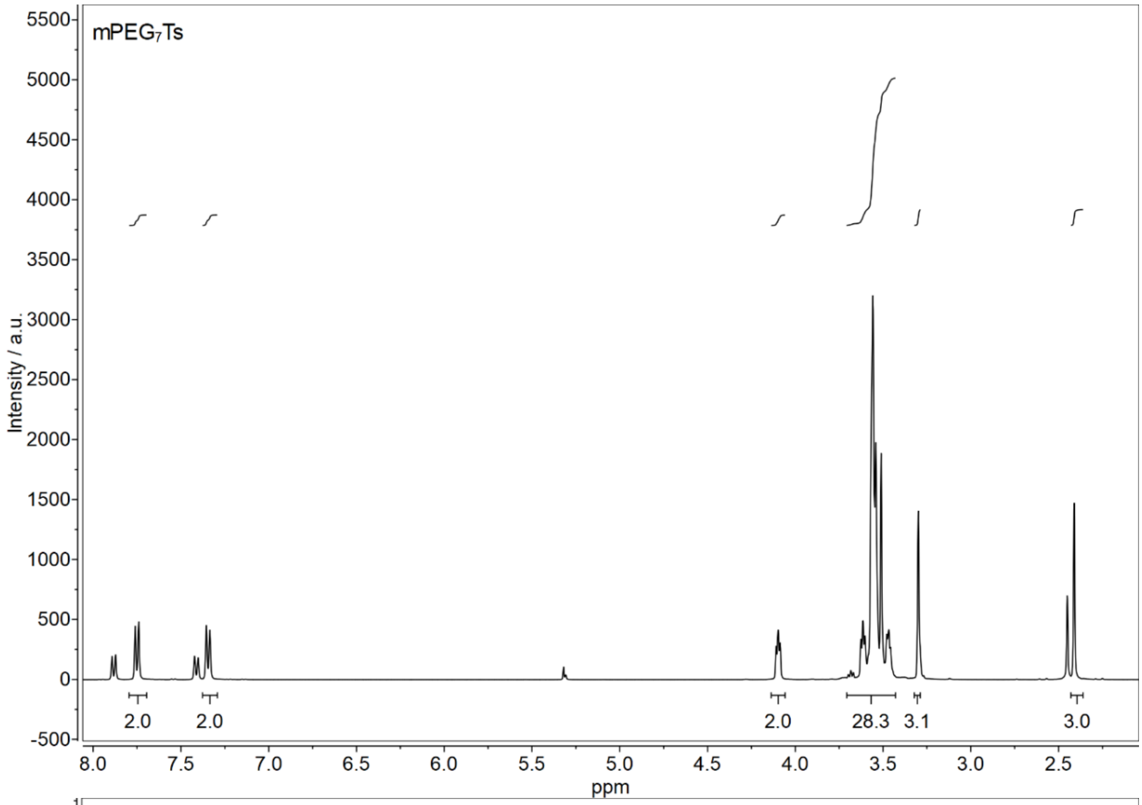
mPEG7Ts
Intensity / a.u.
ppm
2.0
2.0
2.0
28.3
3.1
3.0

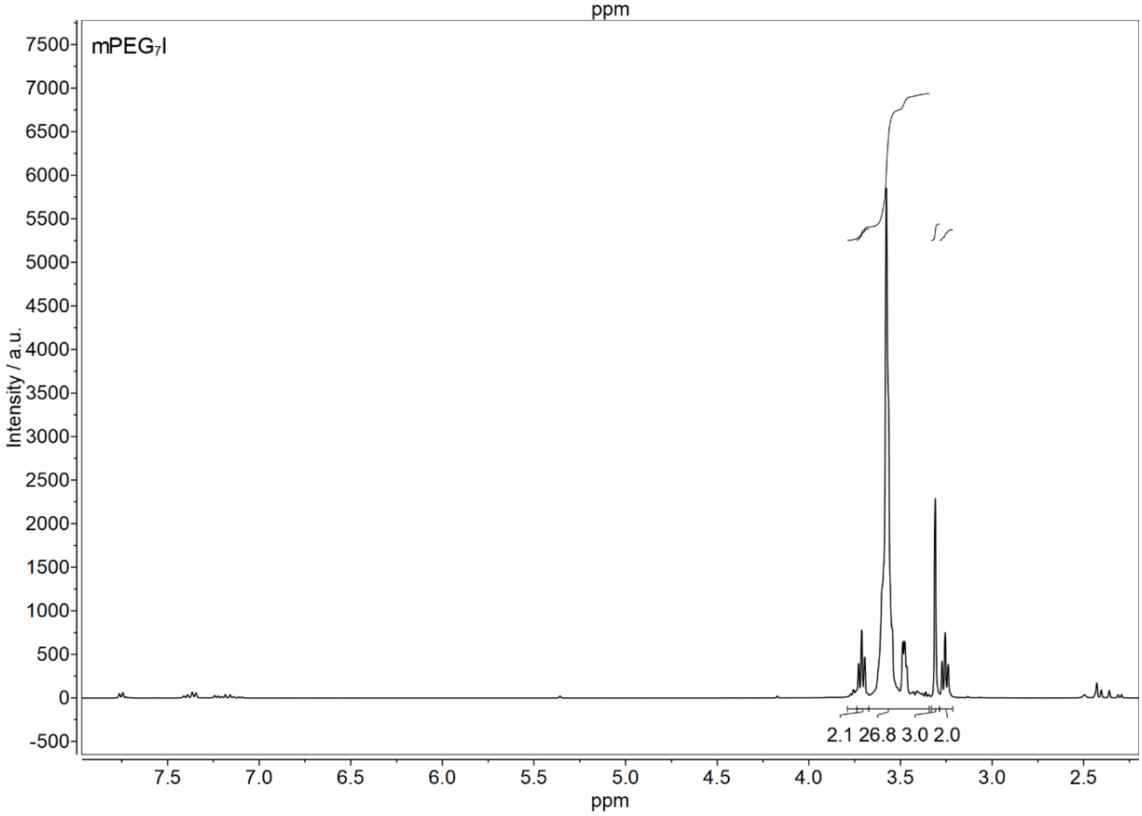
mPEG7I
Intensity / a.u.
ppm
2.1 26.8 3.0 2.0

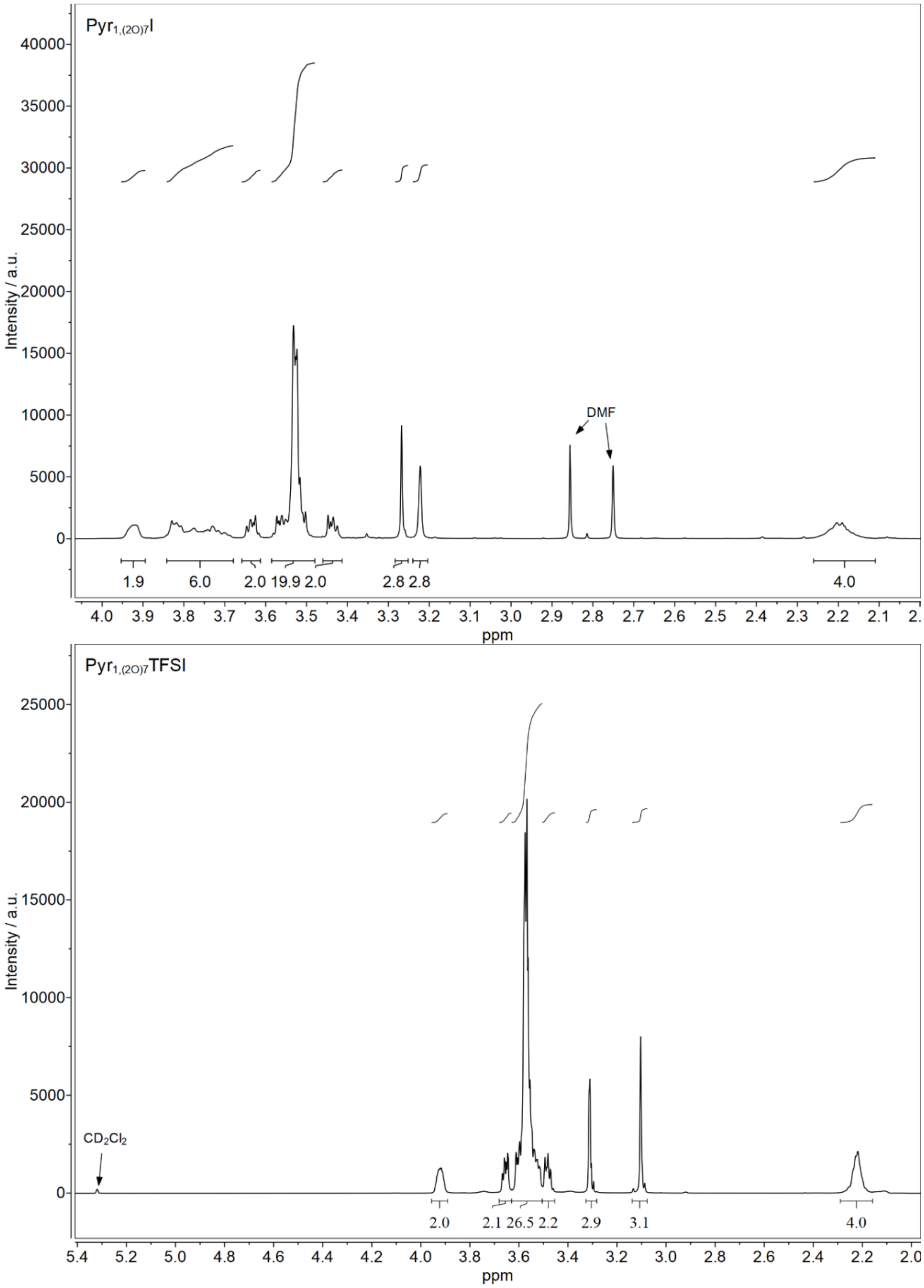

Figure S8. ^{1}H-NMR spectra of all intermediate products and of $Pyr_{1,(2O)7}$TFSI occurring during the $Pyr_{1,(2O)7}$TFSI synthesis path.

3 Molecular Dynamics Simulation Details & Extraction of Transport Model Parameters

Molecular dynamics simulation details – The simulation systems consisted of ten PEO chains with N = 64 monomers each, 64 LiTFSI ion pairs and 32 ion pairs of oligo(ethylene oxide)-based ILs with side chain lengths of one, four, and eight ethylene oxide monomers, corresponding to the experimental molar ratio of 20:2:1. To enhance the ion transport, an elevated temperature of 423 K was employed in the simulations. The force field and all other simulation parameters were identical to those reported previously. [8] The length of the trajectories was in the range of 75-100 ns.

Lithium diffusion along the PEO backbone – To extract τ_1, we first calculated the mean squared number of monomers $\langle \Delta n^2(t) \rangle$ the lithium ions traveled along the backbone. To this end, we consecutively indexed all monomers of a given PEO chain, and for a given lithium ion at a given time, we determined the average index of all monomers of a given strand to which the ion coordinates. Based on these average indices, we computed $\langle \Delta n^2(t) \rangle$, and fitted the curves in the range of 0.1 – 1 ns by a power law $\langle \Delta n^2(t) \rangle = At^{\alpha}$ (Figure S9).

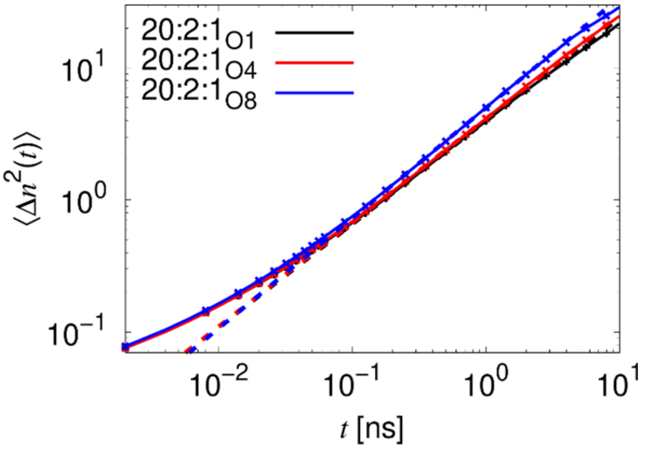

Figure S9. Mean squared numbers of monomers $\langle \Delta n^2(t) \rangle$ the lithium ions traveled along the backbone. The dashed lines represent fits of the form $\langle \Delta n^2(t) \rangle = At^{\alpha}$.

From Figure S9, the diffusion coefficient D_1 can be extracted according to

$$D_1 = \frac{\langle \Delta n^2(t) \rangle}{2t} \tag{7}$$

and τ_1 *via*

$$\tau_1 = \frac{(N-1)^2}{\pi^2 D_1} \tag{8}$$

(see previous papers for details[9]). However, from Figure S9 and the resulting fit parameters in table S3 we note the diffusion along the backbone is still slightly sub-diffusive on the accessible time scales, which affects the determination of D_1. To quantify the net displacement of an ion at a given chain during its entire residence time, we therefore determined D_1 at τ_3, i.e.

$$D_1(\tau_3) = \frac{\langle \Delta n^2(\tau_3) \rangle}{2\tau_3} \tag{9}$$

via extrapolation of the fit curves and computed τ_1 on this basis (Table 1 in the main text).

Table S3. Fit parameters for the curves from Figure S10 *via* the function $\langle \Delta n^2(t) \rangle = At^{\alpha}$.

System	A	α
$20{:}2{:}1_{O1}$	3.958	0.779
$20{:}2{:}1_{O4}$	4.116	0.786
$20{:}2{:}1_{O8}$	4.972	0.834

Cooperative motion of lithium ion and the polymer chain – To characterize the motion of the lithium ions due to the segmental PEO dynamics, we computed the mean squared displacements (MSDs) of all PEO ether oxygen atoms (EOs), of only those EOs that are bound to a lithium ion, and of the respective attached lithium ions (Figure S10). We note that the MSD of the attached lithium ions is slightly lower than that of the bound EOs due to the lack of internal degrees of freedom. The polymer relaxation times for the average and the bound EOs τ_R and τ_2 are then determined *via* the analytical expression

$$g_2(t) = \frac{12\langle \boldsymbol{R}_g^2\rangle}{\pi^2} \sum_{p=1}^{N-1} \frac{\left[1 - \exp\left(-\frac{tp^2}{\tau_2}\right)\right]}{p^2} \tag{10}$$

from the Rouse model[10]. The gyration radii $\langle \boldsymbol{R}_g^2\rangle$ were computed from the polymer structure and not treated as fit parameters, and we used 63 internal modes p for the PEO chains. All resulting parameters are listed in table 1 and table S4.

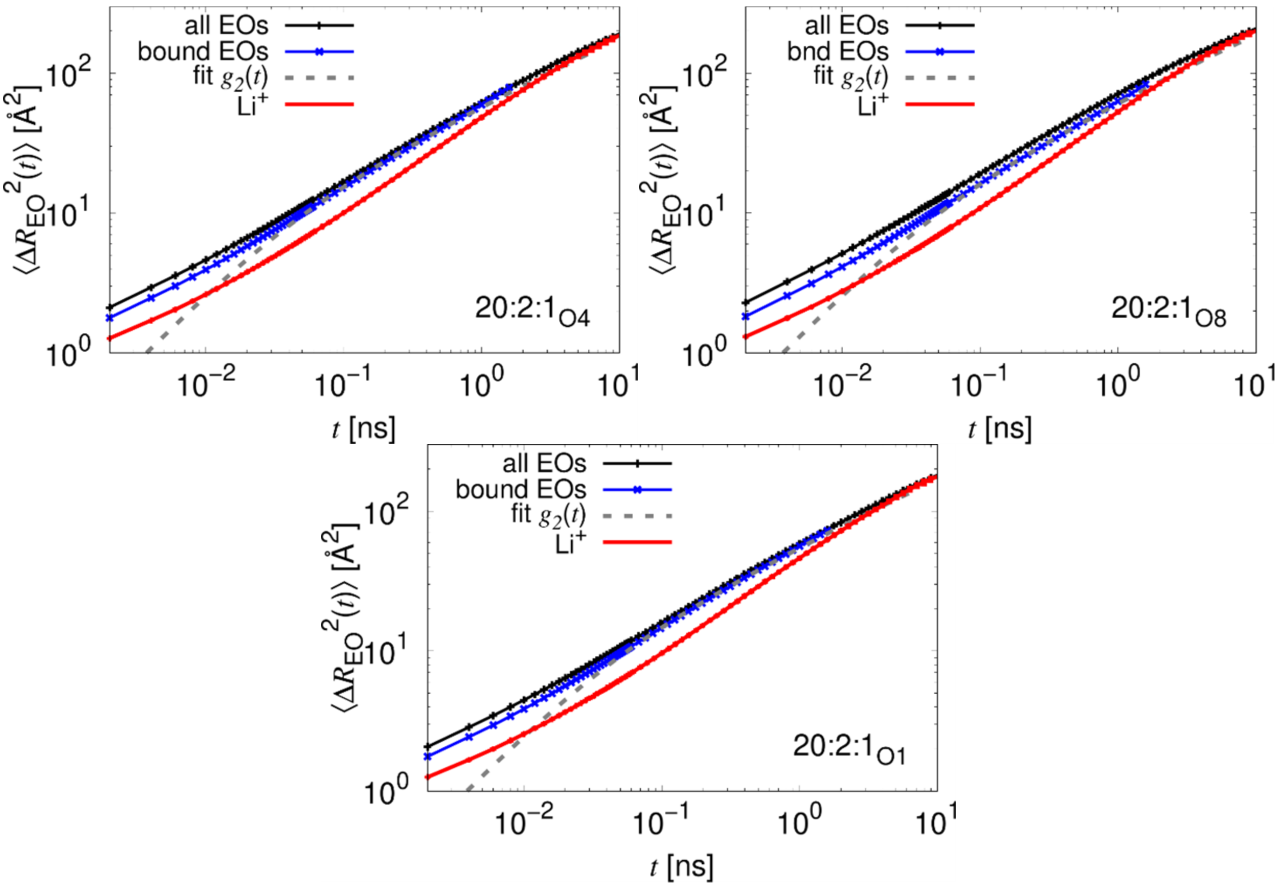

Figure S10. Mean squared displacements (MSDs) of average PEO ether oxygen atoms (EOs) and EOs bound to a lithium ion. The fit *via* the analytical expression of the Rouse model and the MSD of the attached lithium ions is also shown.

Table S4. Mean squared radius of gyration $\langle \boldsymbol{R}_g^2\rangle$ and diffusion coefficient D_{IL} of the lithium ions that are not coordinated to PEO.

System	$\langle \boldsymbol{R}_g^2\rangle$ [Å^2]	DIL [Å^2/ns]
20:2:1$_{O1}$	228	8.7
20:2:1$_{O4}$	238	8.2
20:2:1$_{O8}$	275	8.4

Average residence times at PEO and within the ionic liquid domains – To determine the average residence time of a lithium ion at a given PEO chain, the number of transfer events N_{trans}^{PEO} in which a lithium ion at a PEO chain was either transferred to another chain or to an IL cation has been extracted from the simulations, and τ_3 was computed according to [9]

$$\frac{1}{\tau_3} = \frac{N_{\mathrm{trans}}^{\mathrm{PEO}}}{(1-p_{\mathrm{IL}})t_{\mathrm{sim}}N_{\mathrm{Li^+}}} \tag{11}$$

where t_{sim} is the total simulation time, $N_{\mathrm{Li^+}}$ is the number of lithium ions in the simulation box and division by $(1-p_{\mathrm{IL}})$ accounts for the fraction of lithium ions coordinated to PEO only. Likewise, the average time a lithium ion is coordinated by IL molecules was determined *via* [9]

$$\frac{1}{\tau_{\mathrm{IL}}} = \frac{N_{\mathrm{trans}}^{\mathrm{IL}}}{p_{\mathrm{IL}}t_{\mathrm{sim}}N_{\mathrm{Li^+}}} \tag{12}$$

where $N_{\mathrm{trans}}^{\mathrm{IL}}$ is the number of lithium ion transfers from the IL to PEO.

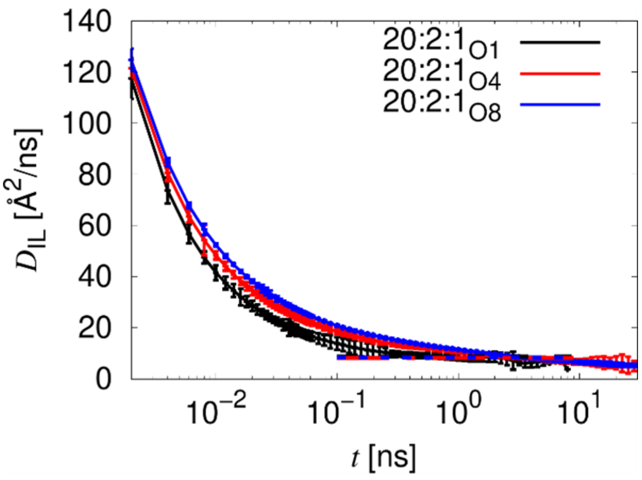

Figure S11. Time-dependent diffusion coefficient of lithium ions not coordinated to PEO. The dashed lines indicate the extracted diffusion coefficients D_{IL}.

Calculation of the lithium ion diffusion coefficients for infinitely long chains – Assuming that the motion of lithium ions at the PEO chain and at the coordinating IL cations is uncorrelated, the lithium diffusion coefficient in the limit of long chains may be calculated as [9]

$$D_{\mathrm{Li}} = (1-p_{\mathrm{IL}})\frac{\langle g_{12}(\tau_3)\rangle}{\tau_3} + p_{\mathrm{IL}}D_{\mathrm{IL}} \tag{13}$$

where the diffusion coefficient of the lithium ions that are not coordinated to PEO D_{IL} was extracted from the simulations (Figure S11 and table S4), and $\langle g_{12}(\tau_3)\rangle$ is the total mean square displacement of a lithium ion during its average residence time τ_3, which may be analytically calculated *via* [9,11]

$$\langle g_{12}(\tau_3)\rangle = \frac{12\langle \boldsymbol{R}_{\mathrm{g}}^2\rangle}{\pi^2}\sum_{p=1}^{N-1}\frac{1}{p^2}\left[1-\frac{1}{p^2\frac{\tau_3}{\tau_{12}}+1}\right] \tag{14}$$

where $\tau_{12}^{-1} = \tau_1^{-1} + \tau_2^{-1}$ is a combined relaxation rate due to lithium diffusion along the backbone and segmental polymer motion. Note that for short chains (i.e. $N < 100$ for PEO), D_{IL} is substantially increased by the center-of-mass motion of the polymer chains, which is irrelevant under experimental conditions (i.e. long chains).

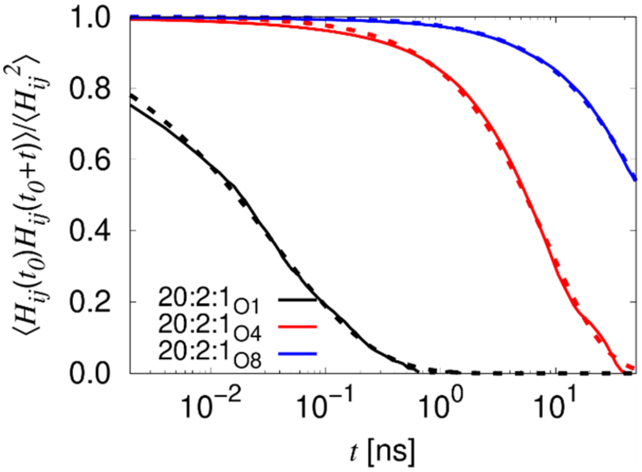

Figure S12. Normalized coordination auto-correlation functions $\langle H_{ij}(t_0)H_{ij}(t_0+t)\rangle/\langle H_{ij}^2\rangle$ as a function of time. The dashed lines indicate fits by a stretched exponential.

Determination of lifetimes of Li⁺/IL cation pairs – To determine the average coordination lifetimes, we defined the function $H_{nm}(t_0)$ that is one if ions *i* and *j* are coordinated at starting time t_0 and zero otherwise, and calculated the correlation function $\langle H_{ij}(t_0)H_{ij}(t_0+t)\rangle/\langle H_{ij}^2\rangle$ [8,12]. Subsequently, the curves were fitted with a stretched exponential

$$\langle H_{ij}(t_0)H_{ij}(t_0+t)\rangle = \langle H_{ij}^2\rangle \exp\left(-\left(\frac{t}{\tau}\right)^{\beta}\right) \quad (15)$$

and the average relaxation times mentioned in the main text were estimated from the according to

$$\langle\tau\rangle = \beta^{-1}\Gamma(\beta^{-1})\tau \quad (16)$$

where Γ is the gamma function. Note that these lifetimes were computed for Li^+/IL cation pairs only, whereas τ_{IL} (Table 1 in the main text) also contains contributions from brief coordinations to the anions or subsequent coordinations to multiple IL cations.

4 References

[1] L. V. N. R. Ganapatibhotla, J. Zheng, D. Roy, S. Krishnan, *Chem. Mater.* **2010**, *22*, 6347.
[2] R. Nölle, K. Beltrop, F. Holtstiege, J. Kasnatscheew, T. Placke, M. Winter, *Mater. Today* **2019**.
[3] G. A. Giffin, A. Moretti, S. Jeong, S. Passerini, *J. Phys. Chem. C* **2014**, *118*, 9966.
[4] G. G. Cameron, *Br. Polym. J.* **1988**, *20*, 299.
[5] a) P. G. Bruce, C. A. Vincent, *J. Electroanal. Chem. Interf. Electrochem.* **1987**, *225*, 1; b) J. Evans, C. A. Vincent, P. G. Bruce, *Polymer* **1987**, *28*, 2324; c) P. G. Bruce, J. Evans, C. A. Vincent, *Solid State Ionics* **1988**, *28-30*, 918.
[6] J. von Zamory, G. A. Giffin, S. Jeremias, F. Castiglione, A. Mele, E. Paillard, S. Passerini, *Phys. Chem. Chem. Phys.* **2016**, *18*, 21539.
[7] W. A. Henderson, S. Passerini, *Chem. Mater.* **2004**, *16*, 2881.
[8] V. Lesch, Z. Li, D. Bedrov, O. Borodin, A. Heuer, *Phys. Chem. Chem. Phys.* **2016**, *18*, 382.
[9] D. Diddens, E. Paillard, A. Heuer, *J. Electrochem. Soc.* **2017**, *164*, E3225.
[10] a) M. Doi, S. F. Edwards, *The theory of polymer dynamics*, Clarendon Press, Oxford, **2007**; b) P. E. Rouse, *J. Chem. Phys.* **1953**, *21*, 1272.
[11] a) D. Diddens, A. Heuer, O. Borodin, *Macromolecules* **2010**, *43*, 2028; b) A. Maitra, A. Heuer, *Phys. Rev. Lett.* **2007**, *98*, 227802.
[12] a) W. Zhao, F. Leroy, B. Heggen, S. Zahn, B. Kirchner, S. Balasubramanian, F. Müller-Plathe, *J. Am. Chem. Soc.* **2009**, *131*, 15825; b) J. R. Keith, S. Mogurampelly, F. Aldukhi, B. K. Wheatle, V. Ganesan, *Phys. Chem. Chem. Phys.* **2017**, *19*, 29134.

5 Author Contributions

J.A performed the experiments and wrote the original draft if not stated elsewhere. D.D performed the simulations and wrote the corresponding part. J.H.T. performed the PFG-NMR measurements. G.B. and M.W. gave advice for the paper structure. E.P. proposed the topic and supervised the work of J.A. and corrected the original draft. All authors discussed the results and reviewed the final manuscript.
